# Self-rankings as a predictor of scientific impact beyond peer review

**DOI:** 10.1038/s43588-026-01039-0

**Published:** 2026-08-24

**Authors:** Buxin Su, Natalie Collina, Garrett Wen, Didong Li, Kyunghyun Cho, Jianqing Fan, Bingxin Zhao, Weijie Su

**Affiliations:** 1 University of Pennsylvania, Philadelphia, PA USA; 2 Yale University, New Haven, CT USA; 3 University of North Carolina at Chapel Hill, Chapel Hill, NC USA; 4 New York University, New York, NY USA; 5 Princeton University, Princeton, NJ USA

**Keywords:** Peer review, Computational science, Policy

## Abstract

Peer review in academic research aims to ensure factual correctness and identify work of high scientific potential, but rapid submission growth has made this increasingly difficult. Here we investigate an additional measure for identifying high-impact research: authors’ rankings of their own submissions to the same artificial intelligence (AI) conference. Grounded in game-theoretic reasoning, we hypothesize that self-rankings are informative because authors understand their work’s conceptual depth and long-term promise. We tested this hypothesis in a large-scale experiment at a leading AI conference. Over more than a year, papers ranked highest by their authors received twice as many citations as their lowest-ranked counterparts, and self-rankings were especially effective at identifying highly cited papers. Self-rankings also outperformed peer-review scores in predicting future citation counts. These findings show that authors’ self-rankings can complement peer review when identifying high-impact AI research.

## Abstract (web summary)

The study finds that authors’ rankings of their own AI conference papers predict later citations better than peer-review scores, suggesting that self-rankings could provide a simple complement to peer review.

## Main

A central objective of peer review is to identify research that may shape a field. This process is under strain in rapidly expanding areas such as artificial intelligence (AI). Submissions to the International Conference on Machine Learning (ICML) rose from 1,676 in 2017 to 12,107 in 2025, while submissions to the Conference on Neural Information Processing Systems (NeurIPS) increased from 3,240 to 21,575 over the same period^1–3^. The pool of experienced reviewers has not expanded at the same rate. Conferences have consequently relied more on graduate and undergraduate reviewers^4^, many without prior publications at these venues, and on language models for reviewing^5^. Tight deadlines and uneven expertise can make it difficult to assess both technical correctness and long-term scientific promise. In a NeurIPS 2021 experiment, approximately half of the accepted papers would have been rejected under a second independent review^6,7^. Such inconsistency makes research with lasting impact harder to identify and raises concern that review outcomes may direct attention away from valuable lines of work.

Less attention has been devoted to structured assessments from authors themselves^8–12^. Authors understand the theoretical foundations, limitations and long-term promise of their own work, including details that may be difficult to convey within a paper or short rebuttal. Reviewers working under tight deadlines may instead emphasize readily quantified gains over broader scientific implications. This concern is particularly relevant in AI, where a marginal benchmark improvement can be easier to assess than a paper’s long-term importance^13^. Author judgments and reviewer judgments may therefore contain different, complementary information.

Soliciting candid author assessments is difficult because authors may inflate absolute evaluations. We therefore elicit comparative rankings from authors with multiple submissions to the same conference, a common situation in AI. Authors cannot label every submission as their best, and under certain conditions this design encourages truthful rankings^11,14,15^. Comparative judgments also ask an easier question than assigning an absolute quality score: which of an author’s own papers is stronger? With the conference organizers’ approval, we asked authors with multiple ICML 2023 submissions to rank them by perceived quality. The experiment collected self-rankings from 1,342 researchers covering 2,592 submissions, together with official review scores and final decisions.

**Extended Data Fig. 1:**
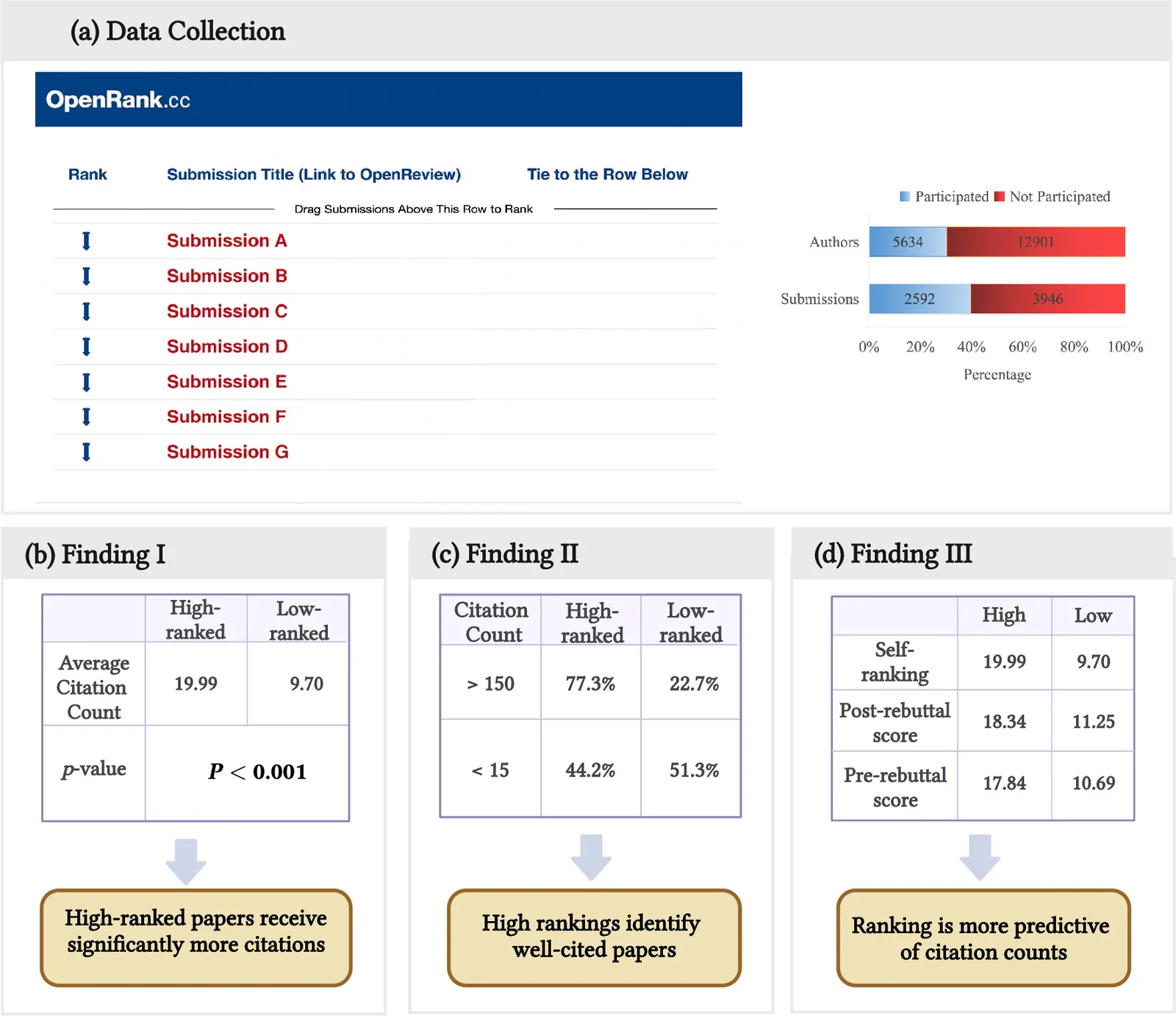
Study design and principal findings. **a**, Illustration of the Phase One survey experiment and summary statistics; the survey interface is shown in Supplementary Figure 1. **b**, Comparison of citation counts between high- and low-ranked submissions (*P* < 0.001, two-sided paired Wilcoxon signed-rank test). **c**, Proportions of high- and low-ranked submissions among well-cited and less-cited papers. **d**, Mean citation counts for submissions partitioned into high- and low-value groups according to self-rankings, pre-rebuttal scores and post-rebuttal scores.

We evaluate whether these rankings predict citations accumulated over 16 months. Citations are widely used, although imperfect, measures of scientific impact^16,17^. Our analysis asks three related questions: whether papers ranked higher by their authors receive more citations on average; whether rankings identify papers in the highly cited tail; and whether rankings add information beyond review scores and final decisions. Papers ranked highest by their authors received about twice as many citations as those ranked lowest, among both accepted and rejected submissions. Among the 22 papers that received more than 150 citations, 17 were ranked highest by at least one author. Self-rankings were also more predictive of future citations than review scores. The associations remained statistically significant after checks involving final decisions, review-score ranges, preprint posting dates and self-citations, and similar patterns appeared with Google Scholar citations and GitHub stars. These results assess whether rankings contain information about later outcomes; they do not imply that citations fully measure quality or that rankings should replace peer review. An overview of the design and principal findings appears in Extended Data Fig. 1.

## Results

The first phase collected author self-rankings through a prereview survey at ICML 2023. OpenReview hosted the review process for 6,538 submissions from 18,535 authors. Immediately after the submission deadline on 26 January 2023, all authors received an official survey implemented through OpenRank.cc. Authors with multiple submissions were asked to rank their papers by perceived quality and answer several questionnaire items; the complete interface is shown in Supplementary Fig. 1. The survey closed on 10 February, before reviews were released to authors. Among 5,634 respondents (30.4% of authors), 1,342 with multiple submissions provided rankings. At least one author ranked each of 2,592 submissions, or 39.6% of all submissions. Multiple submissions were common: 4,505 authors had more than one, and 5,035 submissions had at least one author with multiple submissions. Rankings were therefore available for a substantial share of the conference, although participation was voluntary.

We linked the survey responses to review scores and final decisions from OpenReview. Each paper was evaluated by several reviewers on a 1–10 scale. We call scores assigned before authors responded to reviews pre-rebuttal scores and any revised scores post-rebuttal scores. During rebuttal, authors could address comments and clarify their work without making substantial changes to the submission. The second phase constructed a citation dataset for the ranked submissions using Semantic Scholar. We retrieved papers citing each ranked submission from 23 July 2023 to 22 November 2024 and collected each citing paper’s title, authors and publication date. Matching was complicated by title revisions, changes in author lists and special characters across paper versions. We retained only records for which both the title and author list matched under the procedure described in Methods. The final dataset contained 797 authors and 1,527 unique submissions with valid citation records. Mean citations per paper were 14.73; the median was 4, the 75th percentile was 11 and the maximum was 979. Google Scholar results are reported in Supplementary Section 8.

**Fig. 1:**
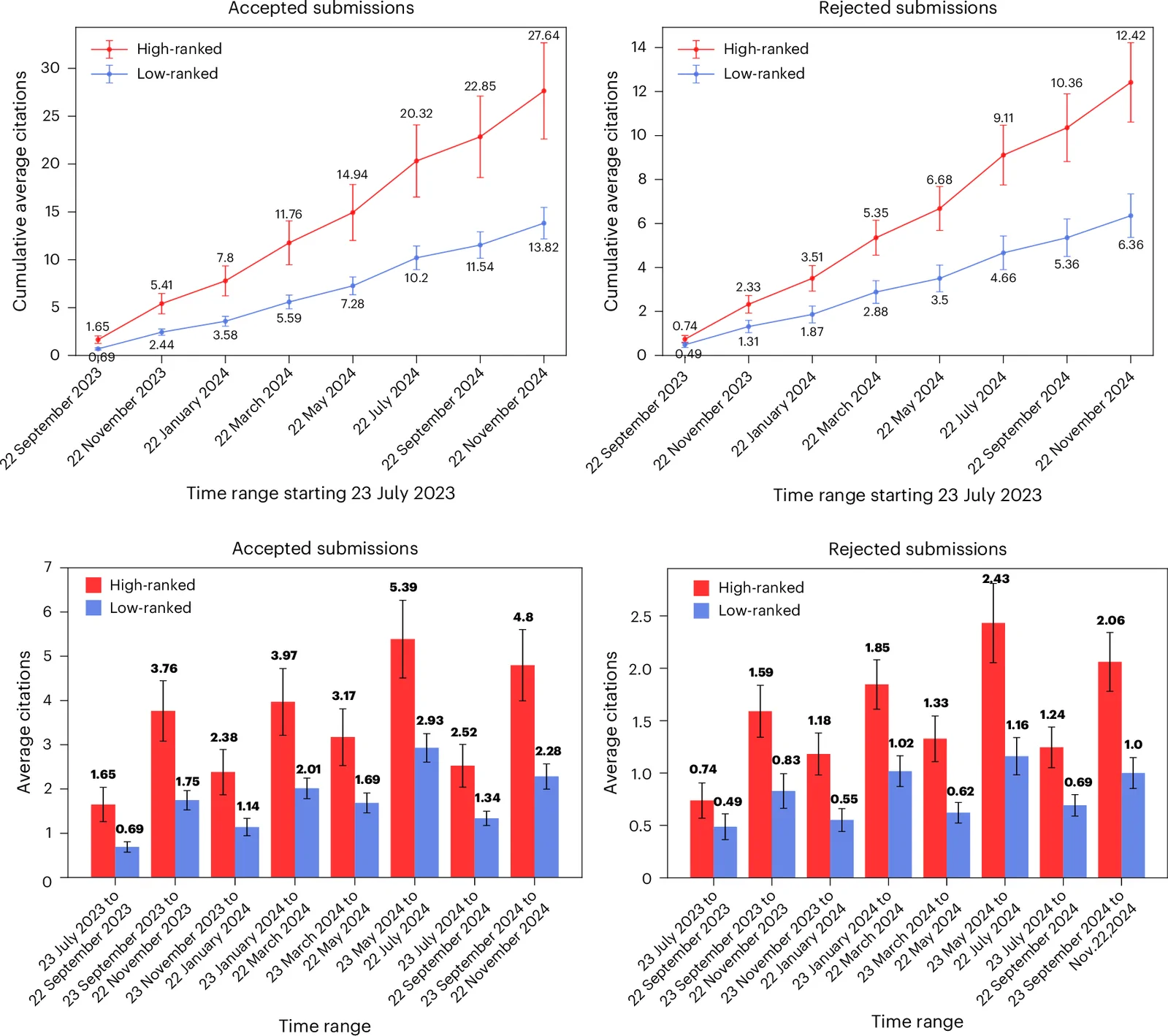
Citation trajectories by author self-ranking and final decision. Citation counts in successive 2-month periods from 23 July 2023 to 22 November 2024, comparing high-ranked papers (red) and low-ranked papers (royal blue), conditional on the final decision. Top: cumulative citations. Bottom: citations accrued within each non-overlapping 2-month period. Left: accepted submissions. Right: rejected or withdrawn submissions. Data are presented as mean values ± standard error of the mean (s.e.m.); each error bar extends one s.e.m. below and above the mean. Each rank group contains *n* = 280 author-level observations in the accepted panels and *n* = 343 in the rejected or withdrawn panels. The unit of analysis is the responding author: within each final-decision group, each author contributes one highest-ranked and one lowest-ranked submission, forming a paired comparison in which the low-ranked submission is the within-author comparator. The stated *n* values count author-level pairs rather than unique papers; a submission can occur in more than one pair when multiple coauthors responded. Supplementary Fig. 9 shows the distributions underlying the top graphs. Source data

The main analysis compared each author’s highest- and lowest-ranked submissions. We defined the top-ranked submission as high-ranked and the bottom-ranked submission as low-ranked. High-ranked papers received 19.99 citations on average, twice the low-ranked mean (*P* < 0.001, two-sided paired Wilcoxon signed-rank test; Extended Data Fig. 1b). We then conditioned on final decisions and time. Among the 797 authors, 280 had multiple accepted submissions and 343 had multiple rejected or withdrawn submissions. ‘Accepted as Poster’ and ‘Accepted as Poster & Oral’ were classified as accepted; ‘Rejected’ and ‘Withdrawn or Desk Rejected’ were classified as rejected. Within each author’s accepted submissions, the highest-ranked paper entered the accepted high-ranked group and the lowest-ranked paper entered the accepted low-ranked group. We applied the same rule to rejected or withdrawn submissions, yielding four groups. This construction compares papers within the same author’s portfolio and within the same broad decision outcome. Figure 1 reports their citation counts over successive 2-month periods.

Cumulative citations grew approximately linearly, with high-ranked papers receiving citations at about twice the rate of low-ranked papers. From 23 July 2023 to 22 November 2024, accepted and rejected high-ranked papers averaged 27.64 and 12.42 citations, respectively, corresponding to 2.00 and 1.95 times the low-ranked means. High-ranked papers also received more citations in every 2-month period (Fig. 1). Four intervals had comparatively high citation volumes: September–November 2023, January–March 2024, September–November 2024 and May–July 2024, in increasing order. These intervals coincided with deadlines for the International Conference on Learning Representations (ICLR), ICML and NeurIPS. Their ordering broadly tracked the corresponding submission volumes of 4,874, 6,538, 7,262 and 12,345 papers, suggesting that conference writing cycles influence when citations appear without explaining the persistent high-versus-low ranking gap.

Rankings beyond the extremes were also informative (Supplementary Fig. 2). Among authors with at least three valid ranked submissions within a decision group, the analysis included 76 accepted and 89 rejected-or-withdrawn author-level rankings. Mean citations for papers ranked first, second, third and last were 41.57, 19.39, 14.32 and 12.83 among accepted submissions, and 9.75, 8.40, 6.34 and 6.18 among rejected or withdrawn submissions. Citation impact therefore declined across ranking positions in both groups, although the first-versus-last contrast had the greatest statistical power.

Self-rankings were especially informative in the highly cited tail of the distribution. Among the 22 submissions with more than 150 citations, 17 (77.27%) were ranked first, 3 were ranked second and 5 were ranked last by at least one author (Extended Data Fig. 1c). One submission was ranked both first and last by different authors, and another was neither. These 22 papers represented approximately 1.44% of the 1,527 submissions, close to the proportion typically selected for oral presentation at major AI conferences.

**Extended Data Fig. 2:**
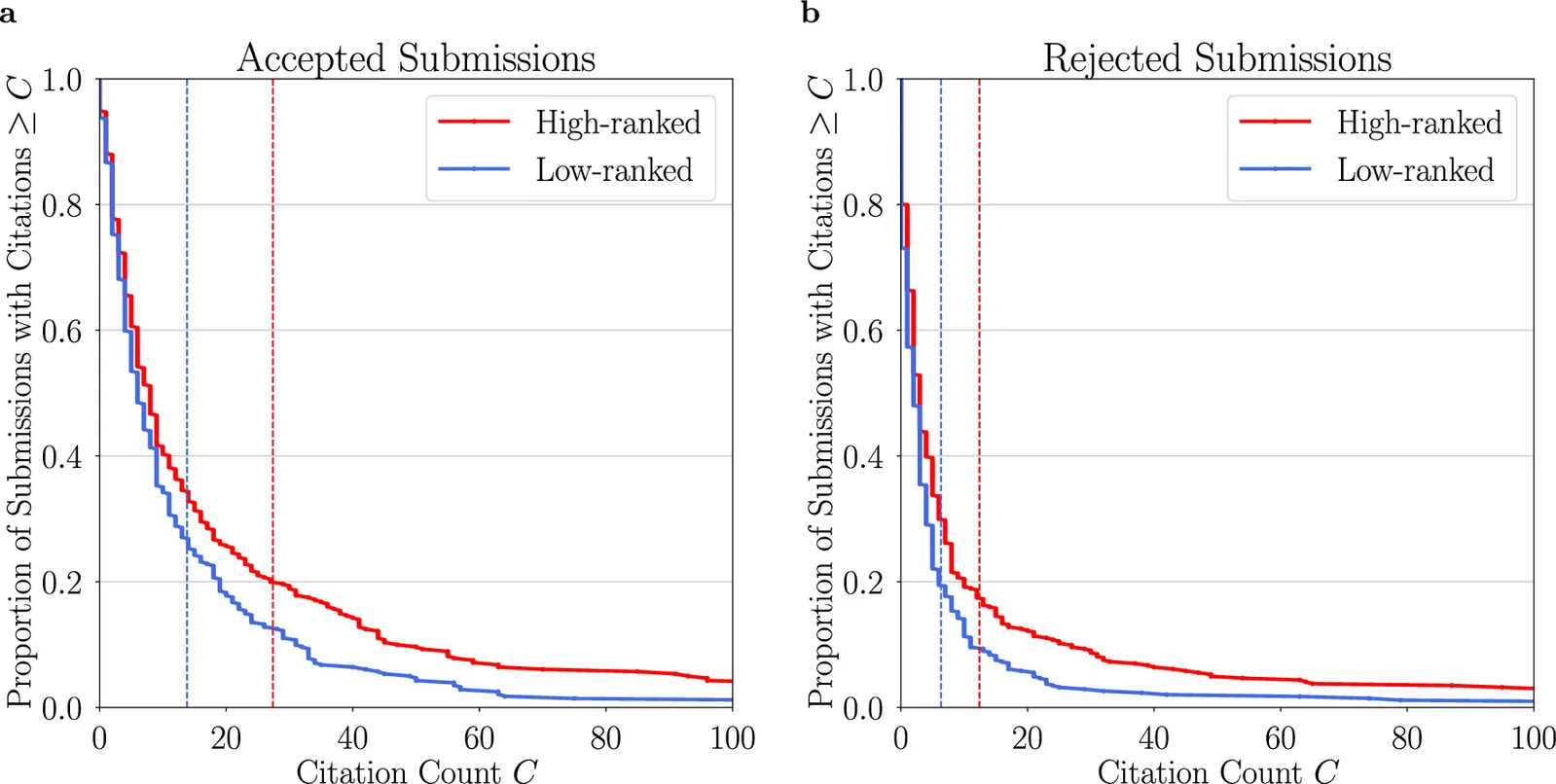
Citation-count distributions by author self-ranking. Empirical complementary cumulative distribution functions for accepted submissions (**a**) and rejected or withdrawn submissions (**b**). At a citation threshold *C*, the vertical coordinate gives the proportion of papers with more than *C* citations. Vertical dashed lines indicate the mean citation counts of the high- and low-ranked groups. Source data

High-ranked papers were more prevalent than low-ranked papers across citation thresholds, with the separation increasing in the upper tail. Extended Data Fig. 2 displays empirical complementary cumulative distributions, which show the fraction of papers exceeding each threshold. The largest gap occurred at 35 citations: 17.14% of high-ranked papers exceeded this threshold compared with 6.79% of low-ranked papers. At 100 citations, the corresponding proportions were 4.29% and 1.43%. Supplementary Table 1 gives the citation percentiles. The lower quartiles were similar, but separation increased in the upper tail. Among accepted submissions, the most-cited high-ranked paper accumulated 979 citations, nearly three times the maximum for a low-ranked paper.

Self-rankings produced a larger and more consistent citation gap than review scores. We compared self-rankings with pre- and post-rebuttal scores among 280 authors with multiple accepted submissions and 343 with multiple rejected or withdrawn submissions. The high-versus-low citation gap was largest for self-rankings in both decision groups (Extended Data Table 1 and Extended Data Fig. 1d). Two-sided paired Wilcoxon signed-rank tests showed statistically significant self-ranking differences among accepted papers (*P* = 6.00 × 10^−3^) and rejected or withdrawn papers (*P* < 0.001). Post-rebuttal scores were also statistically significant among accepted papers (*P* = 6.66 × 10^−3^), but neither score measure was statistically significant among rejected or withdrawn papers. Self-rankings therefore provided the most consistent signal across final decisions. The most-cited paper illustrates this difference: its authors ranked it first, whereas its post-rebuttal score of 5 was lower than their other submissions, and it was accepted as a poster.

**Fig. 2:**
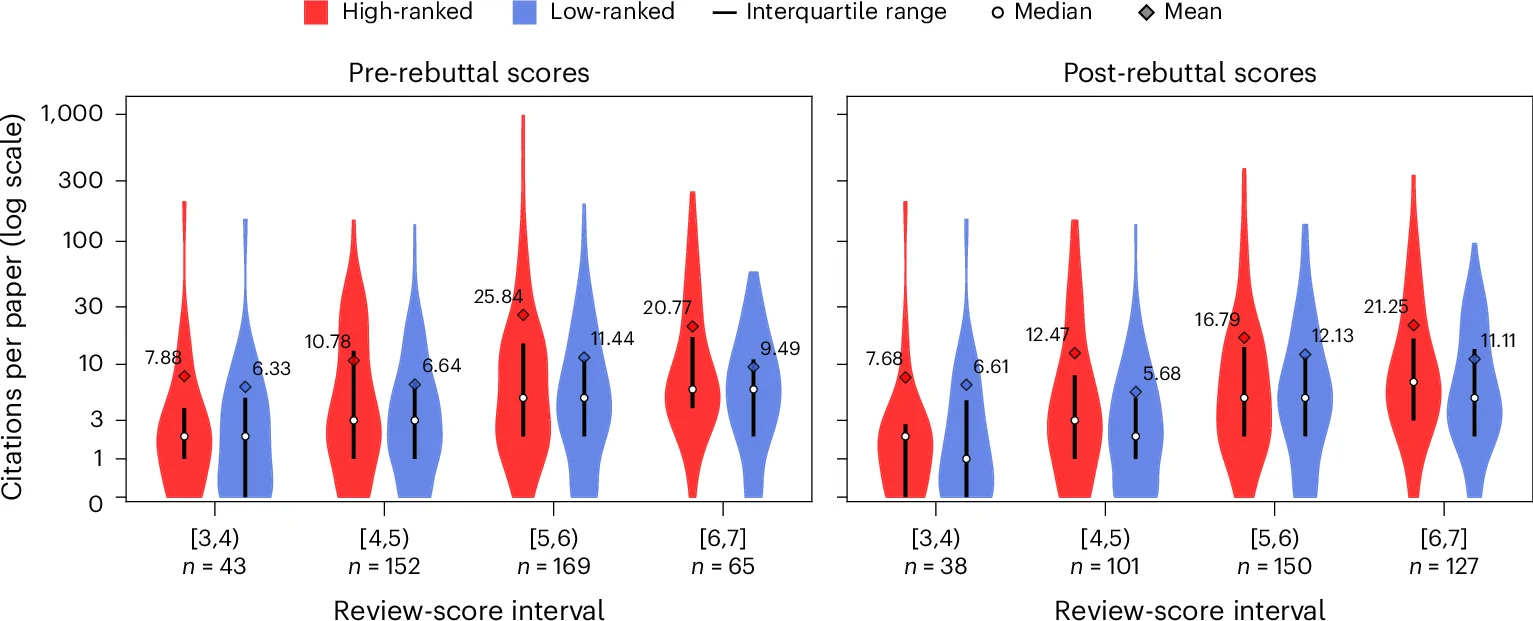
Citation distributions by author self-ranking within review-score intervals. Violin plots show the distributions of citations accumulated from 23 July 2023 to 22 November 2024 for high-ranked (red) and low-ranked (royal blue) papers within ICML 2023 pre- and post-rebuttal review-score intervals. Violin widths represent Gaussian kernel-density estimates of $\log (1+\,\text{citation count}\,)$, with bandwidth selected by Scott’s rule; the vertical-axis tick labels report citation counts on the original scale. Black vertical lines span the 25th to 75th percentiles, white circles denote medians, and diamonds denote arithmetic means on the original citation-count scale; numerical labels give those means. For the pre-rebuttal intervals [3,4), [4,5), [5,6) and [6,7], respectively, each rank group contains *n* = 43, 152, 169 and 65 author-level observations. The corresponding post-rebuttal values are *n* = 38, 101, 150 and 127. The unit of analysis is the responding author: within each score interval, each eligible author contributes one highest-ranked and one lowest-ranked submission, forming a paired comparison in which the low-ranked submission is the within-author comparator. The stated *n* values count author-level pairs rather than unique papers. An author with qualifying submissions in more than one score interval can contribute one pair to each eligible interval, and a submission can occur in more than one pair when multiple coauthors responded. Source data

Self-rankings also separated citation outcomes within narrow review-score ranges. Figure 2 compares high- and low-ranked papers within four similar pre- or post-rebuttal score ranges; Supplementary Fig. 4 gives the corresponding sample sizes. A pair entered a range only when both papers had scores in that interval. Thus, if an author ranked paper *A* above paper *B*, the pair was included in the 5–6 group only when both review scores were between 5 and 6. High-ranked papers generally accumulated more citations within these ranges. Papers with pre-rebuttal scores between 5 and 6 had the highest mean citation count because this group contained the dataset’s two most-cited papers, both ranked first by their authors. The citation gap also widened at higher review scores, indicating that self-rankings add information within score strata. Among the 22 papers exceeding 150 citations, 17 belonged to the high-ranked group and 14 to the high pre-rebuttal-score group; 15 were accepted, while 7 were rejected, including 4 ranked first by at least one author (Extended Data Table 2).

Count regression and holdout prediction confirmed that self-rankings contributed incremental information beyond review variables. The regressions used 2,096 author–submission observations from 780 authors and controlled for review scores and confidence, final decisions, portfolio size, review counts and whether the author supplied a complete ranking. In the fully controlled negative-binomial model, moving from an author’s lowest- to highest-ranked paper was associated with 1.81 times the expected citation count. Repeated holdout prediction used 500 outcome-balanced train–test splits and selected the predicted top quartile of submissions. For all submissions, self-ranking indicators achieved precision 0.318 and recall 0.307, compared with 0.307 and 0.297 for post-rebuttal-score indicators. The gains were larger among rejected or withdrawn papers but small among accepted papers, where self-ranking precision and recall were both 0.261. These results show incremental predictive information rather than sufficient standalone performance. Full specifications and uncertainty summaries are presented in Supplementary Sections 3 and 4.

The ranking–citation relationship persisted across checks for decision, score, portfolio and posting-time confounders. Acceptance and review scores are probably related to both authors’ rankings and later visibility, but stratification showed higher citations for high-ranked papers within both decision and score groups (Figs. 1 and 2 and Extended Data Fig. 2). The within-author design compares papers from the same research portfolio, partially matching on author background, research area and topic. Analyses by portfolio size and co-author ranking agreement, reported in Supplementary Section 2, produced the same overall direction. Posting time was also similar: mean arXiv dates were 4 March 2023 for high-ranked papers and 8 March 2023 for low-ranked papers (Supplementary Fig. 6).

The association also persisted after checks for self-citation and conference publicity. After excluding self-citations, high-ranked papers still received more citations among 649 authors with valid non-self-citation counts (Supplementary Fig. 6). We also examined the 2 months after ICML opened on 23 July 2023. Accepted high-ranked submissions averaged 1.65 citations, more than twice the 0.69 mean for accepted low-ranked submissions. Because subsequent papers that cite conference work usually take months to appear, this early gap is unlikely to result from publicity during the conference. These checks do not eliminate all residual confounding but reduce several specific concerns.

## Discussion

Citations are a practical but incomplete indicator of impact in AI. Unlike settings with a single immediate measure of success, research papers often propose ideas whose value becomes clearer as the community tests, debates and builds on them. A citation indicates that later researchers noticed a paper and considered it relevant enough to connect to their own work, although citations can be positive, neutral or critical. Citation counts can therefore reflect scientific influence without establishing that a paper is correct or intrinsically better. AI papers may also influence products, tools and investment; for instance, heavily cited architectures such as the Transformer underpin many commercial systems^18^. Citation counts nevertheless do not fully measure intrinsic quality, practical value, methodological correctness or benefits outside the research literature.

The pattern was similar for Semantic Scholar citations, Google Scholar citations and GitHub stars, suggesting that the association is not specific to one data source or outcome. Among papers linked to repositories, accepted high-ranked papers averaged 755.15 GitHub stars compared with 347.48 for accepted low-ranked papers; the corresponding rejected-paper means were 89.77 and 33.27. These values are descriptive because repository availability and software use vary across research areas. Longer follow-up and additional conferences are still needed. Related experiments have been conducted at ICML 2024 and 2025 and NeurIPS 2025, but those papers require time to accumulate citations. Another challenge is truthful reporting if rankings influence decisions. Comparative rankings prevent authors from assigning every submission the highest rating, and game-theoretic mechanisms can combine rankings with review scores^11,15^.

Supplementary analyses also place author rankings alongside other comparative signals. A synthetic reviewer-ranking simulation found that reviewer rankings can improve score calibration when reviewer biases are present, especially when reviewer and author rankings are combined. Because its bias and noise parameters were not estimated from the ICML data, this simulation is a mechanism and sensitivity check rather than an empirical estimate of conference-level gains. A separate text-only comparison used a large language model, GPT-4o mini, to rank 263 within-author pairs of accepted papers. The model’s preferred papers averaged 21.69 citations compared with 19.61 for its nonpreferred papers, whereas the author-ranked means for the same pairs were 27.00 and 13.82. This diagnostic analysis is narrower than large-scale large language model-ranking studies and does not establish how stronger models or broader paper pools would perform. It indicates that both the comparative format and the source of the judgment matter.

These results have substantial implications for scholarly evaluation in fast-moving fields. Review systems must distinguish methodological correctness from likely scientific influence. Conventional reviews may favor easily quantified improvements, while authors may have a broader understanding of conceptual contribution and long-term potential. Self-rankings could therefore support tasks such as identifying award candidates or papers that warrant additional scrutiny. They should provide targeted side information rather than replace reviewer judgment.

The purpose of the experiment is to test whether authors possess useful comparative information about the quality or promise of their own submissions. Later citations and GitHub stars are external proxies for downstream scientific impact and community uptake, not complete measures of paper quality and not objectives that conferences should directly maximize. The study covers one conference cycle and only authors with multiple submissions who supplied rankings, limiting generalizability to other venues, fields, years and authors with one submission. Voluntary participation may also select authors whose ranking behavior differs from that of nonparticipants. Conditioning on submitted papers creates another restriction: all analyzed work had already passed an author’s threshold for conference submission, so the results do not describe the broader set of possible research ideas. The observational design cannot eliminate residual confounding; the impact measures are incomplete and time dependent; and author incentives may change if rankings affect decisions. These limitations require cautious interpretation and prospective evaluation before rankings are used more broadly.

ICML 2026 used author self-rankings to identify submissions for additional review-quality scrutiny, based partly on the ICML 2023 findings^19^. After pre-rebuttal scores became available, an isotonic mechanism calculated projected scores consistent with author rankings. Submissions with large discrepancies between projected and original scores were placed in disagreement categories visible to area chairs and senior area chairs, without revealing the discrepancy’s direction. They could then obtain emergency reviews or re-examine existing reviews. Approximately 1,000 submissions were flagged as having high or very high disagreement, about one per area chair. This implementation illustrates how rankings can direct scarce reviewing attention to cases where author information and reviewer scores disagree most.

## Methods

All research was conducted in accordance with relevant ethical regulations. The study protocol was reviewed by the University of Pennsylvania Institutional Review Board, which determined it to be exempt under section 46.104, category 2, of Title 45 of the United States Code of Federal Regulations (IRB Protocol 860005).

### Survey question

Supplementary Fig. 1 shows the complete survey. Authors with multiple submissions ranked their papers by perceived quality by dragging them up or down and answered the other questions shown there. The task was not framed as predicting acceptance, personal contribution or future citations. The survey explained that an isotonic mechanism would take rankings and review ratings as inputs, but that modified ratings would not be used for ICML 2023 decisions. The privacy statement specified that rankings would not be shared with co-authors, reviewers, area chairs, senior area chairs or program chairs and would not affect peer review. Citations are therefore external outcomes used to assess whether perceived-quality rankings contain predictive information, not the criterion authors were asked to rank. Other survey questions are analyzed in Su et al.^20^.

### Extracting citation counts

We submitted each paper title to the Semantic Scholar application programming interface and retained the best-matching record. We extracted its title and author list, then used Levenshtein distance to assess whether it matched the ICML 2023 submission. This distance is the minimum number of single-character insertions, deletions or substitutions needed to transform one sequence into another. Records were treated as the same paper when both title and author-list distances were at most 20. This threshold allowed minor title revisions, added middle names, co-authors added after the OpenReview version and special strings such as ‘Schrödinger’ that can produce small download mismatches.

### Statistics and reproducibility

This observational study analyzed all eligible ICML 2023 survey respondents with multiple submissions, valid author rankings and valid citation matches. No statistical method was used to predetermine sample size. Records without valid rankings or Semantic Scholar matches were excluded because reliable within-author comparisons or citation outcomes could not be obtained; authors with fewer than two remaining eligible submissions could not contribute a paired comparison. No observations were excluded on the basis of their citation outcomes. Exact sample sizes and analysis-specific eligibility criteria are reported in the text, tables and figure legends.

Primary comparisons used two-sided paired Wilcoxon signed-rank tests, with means and standard errors of the mean reported where indicated. Secondary analyses used paired *t*-tests, a two-sided Welch’s *t*-test, Poisson and negative-binomial regressions, repeated holdout prediction and simulations, as described in Supplementary Sections 3–5, 7 and 8. Exact *P* values are reported unless *P* < 0.001.

Because this observational study involved no intervention or allocation, randomization and blinding were not applicable. The experiments were not randomized. The Investigators were not blinded to allocation during experiments and outcome assessment. The study involved neither biological nor technical replicates; the responding author was the unit of analysis for the primary paired comparisons. Privacy-preserving aggregate source data and the publicly archived analysis code support reproducibility.

### Reporting summary

Further information on research design is available in the Nature Portfolio Reporting Summary linked to this article.

## Supplementary information

Supplementary Information Supplementary Sections 1–9, Figs. 1–9 and Tables 1–6.

Reporting Summary

Peer Review File

## Source data

Source Data Fig. 1 Statistical source data: privacy-preserving plotting summary with sample size, mean and s.e.m. of interval and cumulative citation counts for each time interval, ranking group and decision group; no author- or submission-level data are included.

Source Data Fig. 2 Statistical source data: privacy-preserving plotting summary with sample size, arithmetic mean, median, first quartile (Q1) and third quartile (Q3) for high- and low-ranked submissions within each pre- and post-rebuttal score interval; no author- or submission-level data are included.

Source Data Extended Data Fig. 2 Statistical source data: privacy-preserving aggregated empirical complementary cumulative distribution coordinates, group sample sizes and mean citation reference lines; no author identifiers, submission identifiers or author-level pairings are included.

## Data Availability

Individual-level data, including author self-rankings and linked submission and citation records, cannot be made publicly available or shared upon request because of the ICML 2023 Board policy and the privacy terms under which the data were collected (https://openrank.cc/legal/privacy/). No controlled-access mechanism is available for these data. Source data are provided with this paper. These privacy-preserving aggregate summary statistics files contain no individual-level observations, author or submission identifiers or author–submission pairings. No additional individual-level source data are available.

## Extended data

**Extended Data Table 1:** Citation outcomes by author self-ranking and review score

Measure	Group	Accepted: self-ranking	Accepted: post-rebuttal score	Accepted: pre-rebuttal score	Rejected/withdrawn: self-ranking	Rejected/withdrawn: post-rebuttal score	Rejected/withdrawn: pre-rebuttal score
Mean	High	27.64	25.40	23.62	12.42	9.98	9.98
Mean	Low	13.82	17.58	18.11	6.36	9.27	8.70
P value	—	6.00 × 10^−3^	6.66 × 10^−3^	0.112	P < 0.001	0.890	0.556
Maximum	High	979.0	768.0	768.0	253.0	253.0	253.0
Maximum	Low	331.0	979.0	979.0	205.0	251.0	205.0
75th percentile	High	21.25	22.25	18.0	8.0	7.0	7.0
75th percentile	Low	15.0	16.0	16.0	5.0	6.0	6.0

Mean, maximum and 75th-percentile citation counts from 23 July 2023 to 22 November 2024 are reported for high and low groups defined within author portfolios by self-ranking, post-rebuttal score or pre-rebuttal score, separately for accepted and rejected or withdrawn submissions. *P* values are from two-sided paired Wilcoxon signed-rank tests.

**Extended Data Table 2:** Classification of 22 highly cited submissions

Self-ranking	Pre-rebuttal score	Post-rebuttal score	Final decision
17 (high-ranked)	14 (high-scored)	14 (high-scored)	15 (accepted)
5 (low-ranked)	6 (low-scored)	8 (low-scored)	7 (rejected)

Counts show how many submissions with more than 150 citations were classified as high or low by author self-ranking, pre-rebuttal score or post-rebuttal score, and how many were accepted or rejected. Within the self-ranking and score columns, categories are not necessarily mutually exclusive: a submission can be classified as high by one responding coauthor and low by another, or as neither extreme. For pre-rebuttal scores, one submission appears in both the high and low groups and three appear in neither.

## Code availability

The code used to generate the results is publicly available via GitHub at https://github.com/BuxinSu/ICML_2023_Citationand is also available via Zenodo at https://doi.org/10.5281/zenodo.21501732 (ref. ^21^). Version 1.0.0 is released under the Creative Commons Attribution 4.0 International (CC BY 4.0) license.
