# Supplementary material for: Self-rankings as a predictor of scientific impact beyond peer review
Source: Nat Comput Sci. 2026 Aug 24;6(9):939–44. doi: 10.1038/s43588-026-01039-0 (PMC13593569; doi:10.1038/s43588-026-01039-0)
Supplement: Supplementary file 1 [file 43588_2026_1039_MOESM1_ESM.pdf]

nature computational science

Supplementary information

https://doi.org/10.1038/s43588-026-01039-0

Self-rankings as a predictor of scientific impact beyond peer review

In the format provided by the authors and unedited

Contents

Supplementary Section 1. Survey interface . . . 2
Supplementary Section 2. Additional details of the main citation analyses . . . 2
Supplementary Section 3. Additional count-regression analysis . . . 5
Supplementary Section 4. Additional holdout prediction analysis . . . 6
Supplementary Section 5. Additional reviewer-ranking simulation . . . 7
Supplementary Section 6. LLM-based pairwise rankings . . . 9
Supplementary Section 7. Additional confounder checks . . . 10
Supplementary Section 8. Alternative impact metrics . . . 11
Supplementary Section 9. Distributional view of cumulative citations . . . 14

Supplementary Section 1. Survey interface

The survey was sent on January 26, 2023, and closed on February 10, 2023. Among the 1,342 authors with multiple submissions who provided valid rankings, the average response time was approximately 4 days; 25% responded within 6.21 hours and 90% responded within 8.79 days.

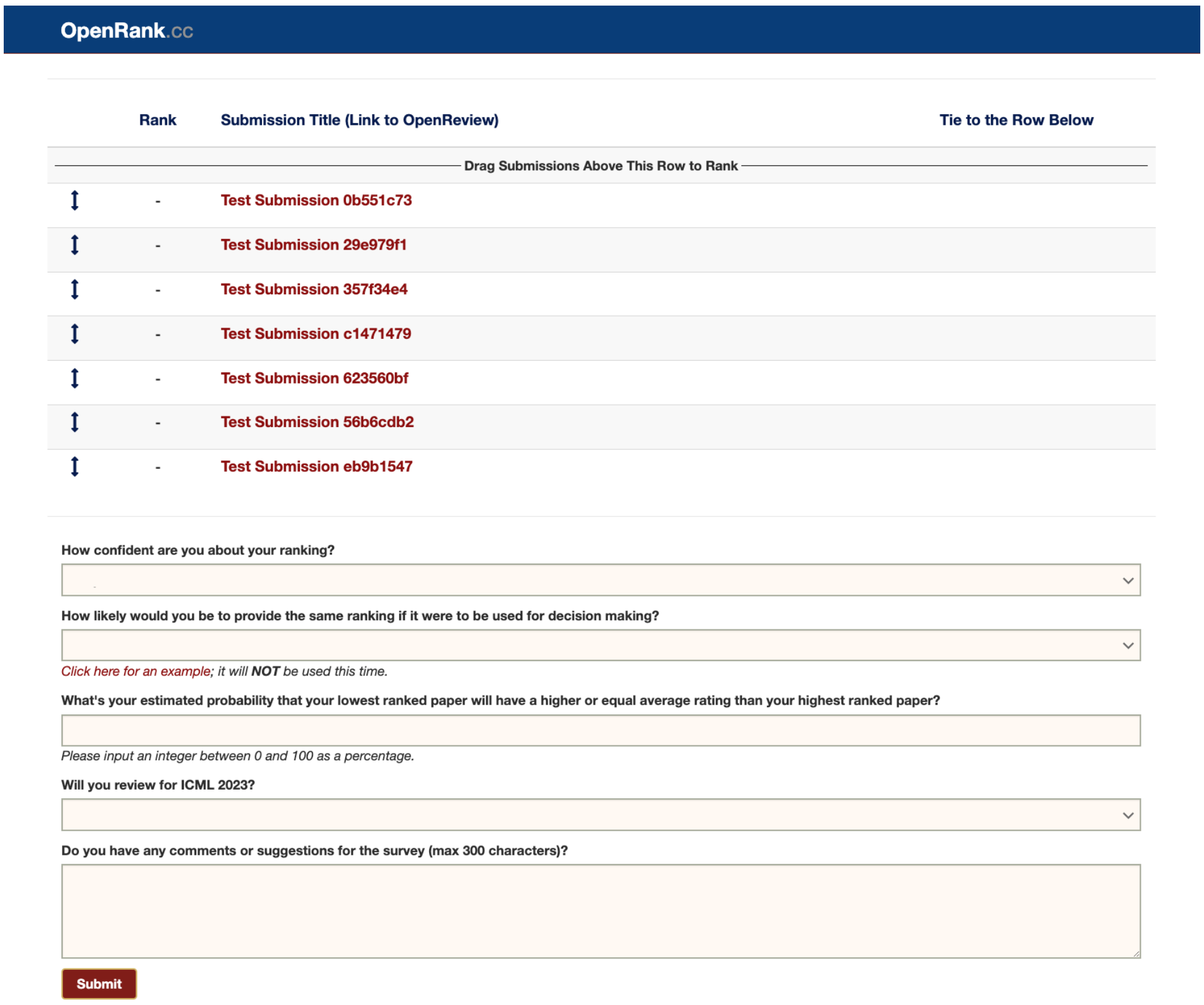

Supplementary Figure 1: Author survey interface. The screenshot shows the instructions and drag-and-drop interface used by authors to rank their ICML 2023 submissions.

Supplementary Section 2. Additional details of the main citation analyses

Supplementary Figure 2 extends our analysis beyond the comparison of only the top- and bottom-ranked submissions. We consider authors with at least three valid ranked submissions within each final-decision group: 76 author-level rankings for accepted submissions and 89 for rejected or withdrawn submissions. For accepted submissions, the first-, second-, third-, and last-ranked bars are computed from 76 author-paper observations each, corresponding to 69, 70, 71, and 70 unique submissions, respectively. For rejected or withdrawn submissions, the four bars are computed from 89 author-paper observations each, corresponding to 86, 89, 85, and 84 unique submissions. As shown in Supplementary Figure 2, average citation counts decline from first to second to third to last in both final-decision strata. Adjacent ranks are noisier and do not all differ significantly, so this analysis should be interpreted as descriptive evidence that rankings beyond the first and last positions contain useful information at the group level.

Supplementary Table 1 reports the citation counts for high- versus low-ranked submissions, conditional on their final decisions. These results complement Extended Data Figure 2 by

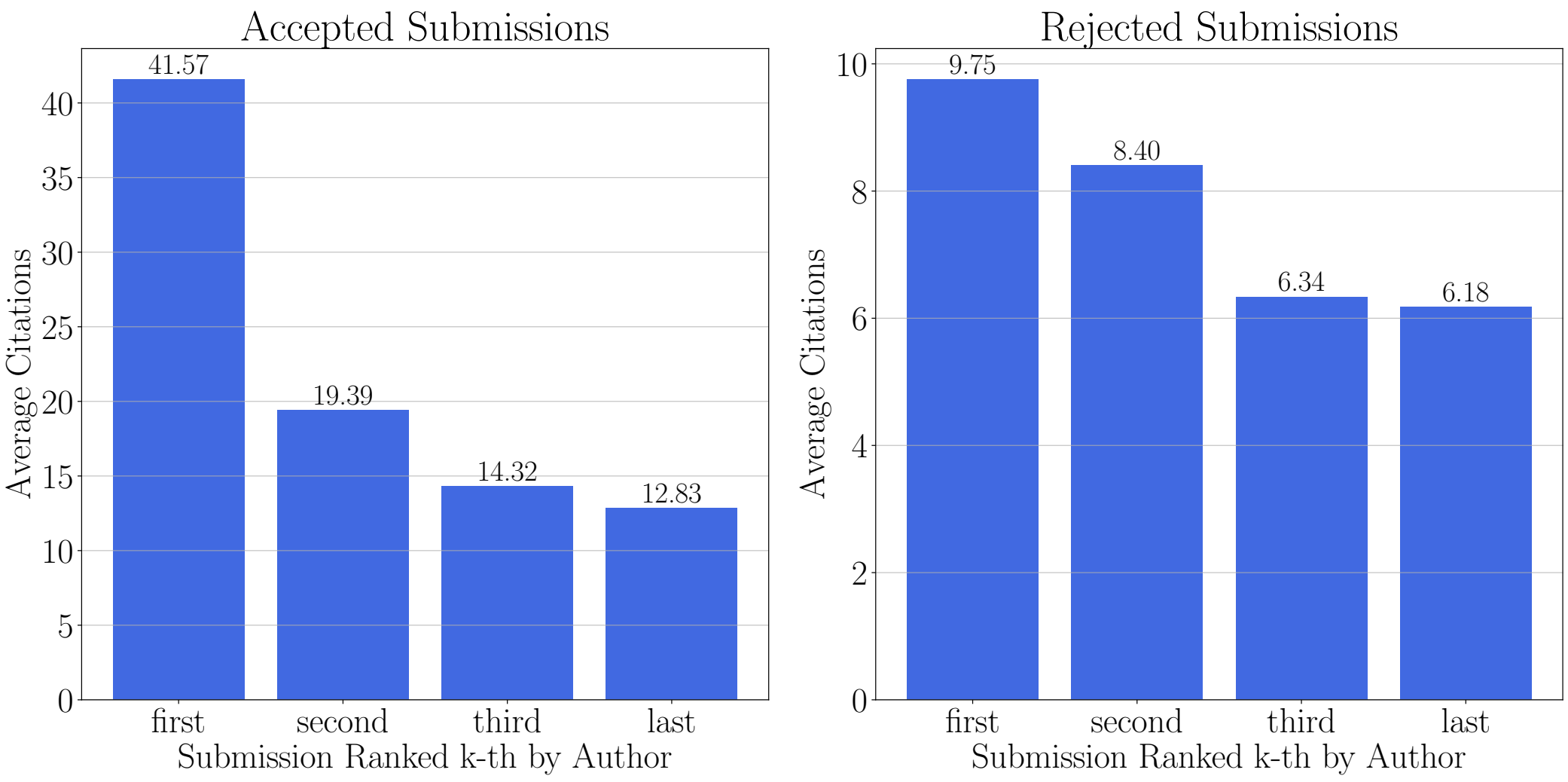

Supplementary Figure 2: Author-ranked citation counts by final decision. Bars show mean Semantic Scholar citations accumulated from July 23, 2023, to November 22, 2024, for first-, second-, third-, and last-ranked submissions; the left panel contains accepted submissions and the right panel contains rejected or withdrawn submissions. Error bars show standard errors of the mean (SEM). Each rank contains $n = 76$ responding-author–paper observations in the accepted panel and $n = 89$ responding-author–paper observations in the rejected or withdrawn panel. Individual author-level observations are not displayed because the underlying ranking data are private; the bars, SEMs and exact sample sizes provide privacy-preserving summaries. No statistical comparison is displayed in this figure.

providing exact statistics.

	Citation Count for Accepted Papers		Citation Count for Rejected/Withdrawn Papers	
	high-ranked	low-ranked	high-ranked	low-ranked
Mean	27.64	13.82	12.42	6.36
p-value	6.00×10^{-3}		< 0.001	
Max	979.0	331.0	253.0	205.0
75th percentile	21.25	15.0	8.0	5.0
50th percentile	8.0	6.0	3.0	2.0
25th percentile	3.0	3.0	1.0	0.0
Number of authors	280		343	

Supplementary Table 1: Semantic Scholar citation summary by author ranking and final decision. Columns separate high- and low-ranked submissions within the accepted and rejected or withdrawn groups. Rows report the mean, two-sided paired Wilcoxon signed-rank P-value, maximum, percentiles and exact number of author-level pairs for citations accumulated from July 23, 2023, to November 22, 2024.

We further examined whether the high- versus low-ranked comparison was driven by authors with larger submission portfolios. We grouped authors by the number of valid ranked submissions, from 2 to 9, and repeated the within-author comparison separately within each portfolio-size group. As shown in Supplementary Figure 3, the effect was already present among authors with exactly two ranked submissions, where highest-ranked papers received 17.32 citations on average compared with 10.48 for lowest-ranked papers, and among authors with exactly

three ranked submissions, where the corresponding averages were 21.33 and 7.64. These two groups account for 87.8% of authors in the grouped analysis, indicating that the overall result is not driven by authors with unusually large submission portfolios. The same direction persisted for authors with 4–9 ranked submissions, although these groups were smaller and noisier. Aggregating all authors with 2–9 ranked submissions, the highest-ranked papers received 19.99 citations on average, compared with 9.73 for the lowest-ranked papers ($P < 0.001$, two-sided paired Wilcoxon signed-rank test).

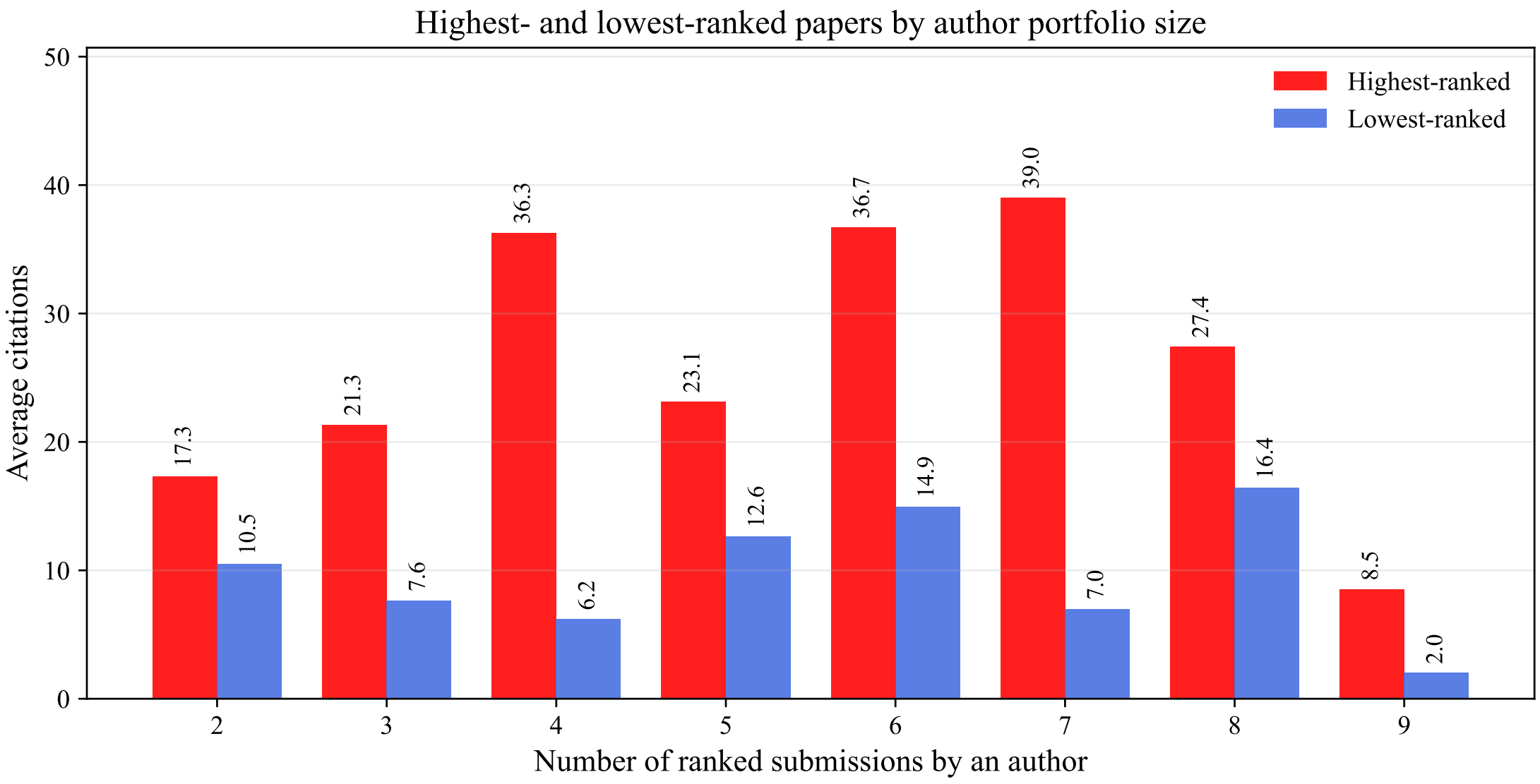

Supplementary Figure 3: Citation counts by author portfolio size. Red and royal-blue bars show mean Semantic Scholar citations for the highest- and lowest-ranked submissions, respectively, within groups defined by the number of valid ranked submissions from the same author; labels above the bars give the corresponding means. Error bars show standard errors of the mean (SEM). For portfolio sizes 2 through 9, each rank group contains, respectively, $n = 516$, $n = 178$, $n = 47$, $n = 26$, $n = 13$, $n = 3$, $n = 5$ and $n = 2$ responding-author observations. Within each portfolio-size group, the same authors contribute one highest- and one lowest-ranked submission, so these values also give the numbers of paired comparisons. Two-sided paired Wilcoxon signed-rank tests comparing the two ranks within portfolio sizes 2 through 9 yielded, respectively, $P < 0.001$, $P < 0.001$, $P = 0.035$, $P = 0.647$, $P = 0.084$, $P = 0.250$, $P = 0.100$ and $P = 0.500$. Individual author-level observations are not displayed because the underlying ranking data are private.

Because some submissions were ranked by multiple coauthors, we also examined whether the high- versus low-ranked comparison was sensitive to coauthor disagreement. The main analysis is author-level: a submission is high-ranked or low-ranked relative to a particular responding author's own portfolio. In the final citation dataset, 466 of the 1,527 unique submissions were ranked by more than one author. Among the 1,248 submissions that appeared in at least one author-level high- or low-ranked group, 126 were high/low conflicts, meaning that the same submission was high-ranked by one responding author and low-ranked by another. Removing these submissions did not weaken the result. As shown in Supplementary Table 2, high-ranked papers still received 20.82 citations on average, compared with 9.11 for low-ranked papers. Because the anonymized analysis data do not reliably identify primary or corresponding authors, we also performed a random paper-level de-duplication check: for each submission ranked by multiple responding authors, we randomly retained one author's ranking and discarded the re-

maining rankings for that submission. After removing authors with only one remaining ranked submission, this result again persisted. Across 1,000 random repetitions, high-ranked papers received 21.13 citations on average, compared with 9.70 for low-ranked papers; in every repetition, the high-ranked mean exceeded the low-ranked mean and the paired test was significant at $p < 0.01$. These robustness checks suggest that coauthor disagreement does not drive the main high-versus-low citation result.

Robustness check	Number of author pairs	High-ranked mean	Low-ranked mean	p-value
Original author-level comparison	797	19.89	9.75	< 0.001
Use only papers with consistent coauthor labels	631	20.82	9.11	< 0.001
Randomly keep one ranking per submission	543	21.47	8.99	< 0.001
Random assignment, 1,000 repetitions	529.36	21.13	9.70	< 0.001

Supplementary Table 2: Citation comparisons under alternative handling of coauthor rankings. Each row reports a paired within-author comparison under the stated inclusion or de-duplication rule. The random-assignment row summarizes 1,000 repetitions by reporting the mean number of author pairs, mean citation counts and median paired-test P-value.

Supplementary Section 3. Additional count-regression analysis

As an additional robustness analysis, we fitted count regressions for citation counts using the author-submission pair as the unit of analysis. This unit is appropriate because the same submission can receive different ranks from different responding coauthors. For each author, we normalized the self-ranking to range from 0 for the lowest-ranked retained submission to 1 for the highest-ranked retained submission. Authors whose retained submissions were all tied in rank do not provide a highest-versus-lowest contrast, so they were excluded from this analysis. The resulting regression sample contains 2,096 author-submission observations from 780 authors and 1,502 unique submissions.

We fitted Poisson and negative-binomial regressions with increasingly rich controls, including review scores and confidences, final-decision indicators, portfolio size, review counts, and full-ranking status. For count-model effect sizes, Supplementary Table 3 reports the incidence-rate ratio (IRR), $\exp(\widehat{\beta})$, for moving from an author's lowest-ranked retained paper to the highest-ranked retained paper. We assess model fit using McFadden's pseudo-R^2, the Akaike information criterion (AIC), and the Bayesian information criterion (BIC). Because citation counts are highly overdispersed, we interpret the negative-binomial regressions as the primary count-model specification.

The self-ranking coefficient remains positive across all specifications. In the fully controlled negative-binomial model, moving from an author's lowest-ranked paper to the highest-ranked paper is associated with 1.81 times as many expected citations, holding review scores, review confidence, final decision, portfolio size, review counts, and full-ranking status fixed. Adding self-ranking also improves model fit relative to the same specification without self-ranking, reducing AIC by 65.3 and BIC by 59.6 in the fully controlled negative-binomial model. The incremental pseudo-R^2 is modest, indicating that self-rankings provide complementary predictive information rather than a complete explanation of future citation impact.

	Poisson				Negative Binomial			
Controls	IRR	R^2	AIC	BIC	IRR	R^2	AIC	BIC
No controls	2.050	0.036	84346.9	84358.2	2.057	0.007	14613.0	14629.9
Review scores/confidence	1.883	0.088	79821.1	79855.0	1.880	0.015	14500.0	14539.5
+ final decision	1.790	0.141	75188.4	75239.3	1.794	0.020	14429.2	14485.7
+ portfolio/review-count controls	1.786	0.150	74400.6	74474.0	1.806	0.022	14407.2	14486.3

Supplementary Table 3: Count-regression specifications for citation counts. Columns report incidence-rate ratios (IRRs), McFadden pseudo-R^2, Akaike information criterion (AIC) and Bayesian information criterion (BIC) for Poisson and negative-binomial models. The IRR represents the multiplicative change associated with moving from an author's lowest- to highest-ranked retained paper. Successive rows add review, final-decision, portfolio and review-count controls as defined in the text.

Supplementary Section 4. Additional holdout prediction analysis

To distinguish explanatory association from predictive performance, we conducted holdout prediction analyses using separate training and holdout sets [1]. For each author portfolio within each paper group, we coded whether a submission was the author's highest-ranked submission, lowest-ranked submission, or neither. We then constructed analogous reviewer-score indicators for whether the submission had the highest or lowest pre-rebuttal score, and whether it had the highest or lowest post-rebuttal score, within the same author portfolio and paper group. Score ties with no within-author contrast were not coded as either high or low.

We considered a submission-level top-quartile citation prediction task for all submissions, accepted submissions, and rejected/withdrawn submissions. We aggregated author-level high/low indicators to one row per submission, including indicators for high-only, low-only, and conflicting high/low labels when different responding authors assigned different extreme labels. We then used 500 outcome-balanced random splits, with 70% of submissions used for training and 30% held out for evaluation. In each training set, we fitted logistic regressions using highest/lowest self-ranking indicators, highest/lowest pre-rebuttal score indicators, or highest/lowest post-rebuttal score indicators. In each holdout set, we selected the top 25% of submissions by predicted probability and computed precision and recall.

Task	Metric	Self-ranking	Post-rebuttal score	Pre-rebuttal score
All submissions: top 25%	Precision	0.318	0.307	0.289
All submissions: top 25%	Recall	0.307	0.297	0.279
Accepted submissions: top 25%	Precision	0.261	0.223	0.245
Accepted submissions: top 25%	Recall	0.261	0.223	0.245
Rejected/withdrawn submissions: top 25%	Precision	0.298	0.230	0.233
Rejected/withdrawn submissions: top 25%	Recall	0.292	0.225	0.228

For each reported metric, self-ranking exceeds both score baselines with two-sided paired Wilcoxon $P < 0.001$.

Supplementary Table 4: Holdout prediction metrics for author rankings and reviewer scores. Rows report precision and recall for submission-level top-quartile citation prediction in each paper group. Columns compare self-ranking indicators with analogous post- and pre-rebuttal score indicators. The P-values compare split-level metrics using two-sided paired Wilcoxon signed-rank tests across the repeated holdout splits.

The holdout prediction results support a more targeted version of the predictive claim. Highest/lowest self-ranking indicators outperform both highest/lowest post-rebuttal score indicators

and highest/lowest pre-rebuttal score indicators in all three paper groups. Among all submissions, precision increases from 0.307 for post-rebuttal score extremes and 0.289 for pre-rebuttal score extremes to 0.318 for self-ranking, and recall increases from 0.297 and 0.279 to 0.307. The gains are larger among rejected/withdrawn submissions: precision increases from 0.230 and 0.233 to 0.298, and recall increases from 0.225 and 0.228 to 0.292. These results indicate that author self-rankings provide useful out-of-sample predictive information for identifying top-cited papers, while also showing that the gains vary across paper groups.

Because the outcome is top-quartile citation status and each model selects the top 25% of held-out submissions, a no-information selector has expected precision and recall of approximately 0.25. For all submissions, the self-ranking model achieves a precision of 0.318 (standard deviation (SD) = 0.034) and a recall of 0.307 (SD = 0.033); these SDs summarize variation across the 500 held-out splits. Thus, self-ranking provides a nontrivial enrichment over chance. This interpretation is important because the deliberately parsimonious model uses only indicators for highest-ranked, lowest-ranked, and conflicting author labels. This construction places self-rankings and reviewer-score extremes on comparable footing, but it is not intended to be an optimized prediction system incorporating paper content, research area, author history, or other potential predictors of citations. The absolute performance therefore remains modest.

The accepted-submission result illustrates an important limitation. For accepted submissions, self-ranking precision and recall are both 0.261 (SD = 0.061), so the standalone predictive gain is very small. One plausible explanation is range restriction induced by conditioning on acceptance: accepted papers have already passed a reviewer- and Area-Chair-based selection process and constitute a more homogeneous set in terms of perceived quality, review scores, and publication visibility. Within this restricted set, the highest/lowest self-ranking indicators alone provide little ability to distinguish which individual papers will enter the top citation quartile. We regard this as a plausible interpretation rather than a causal conclusion.

Accordingly, Supplementary Table 4 does not support using self-rankings as a standalone rule for predicting citations or making acceptance decisions. Rather, it supports using them as complementary side information, for example in disagreement-based triage to identify submissions whose reviews may warrant additional scrutiny, especially below the acceptance threshold.

Supplementary Figure 4 reports the number of authors with multiple ranked submissions, grouped by the review scores of their papers. This summarizes the sample sizes for each group shown in Figure 2 of the main text.

Supplementary Section 5. Additional reviewer-ranking simulation

We further study whether rankings induced by reviewers' evaluations can also be used to improve peer review. Our citation-analysis dataset does not contain reviewer-level assignments or reviewer-level rankings, so we cannot directly apply reviewer-generated rankings to the observed ICML 2023 citation data. Instead, we implemented a synthetic simulation following the setup of Wang and Shi [2].

In this simulation, each paper has a latent quality and each reviewer has a reviewer-specific bias and score-noise level. Reviewers generate noisy raw scores and rankings of their assigned papers according to a Plackett–Luce model, and the final review scores are obtained by isotonic regression so that they are consistent with the reviewer-side rankings. We generated 6,538 synthetic submissions and compared four estimators of latent paper quality: average review scores, scores adjusted using reviewer rankings, scores adjusted using author rankings, and scores adjusted using both reviewer and author rankings. We evaluated the estimators in a

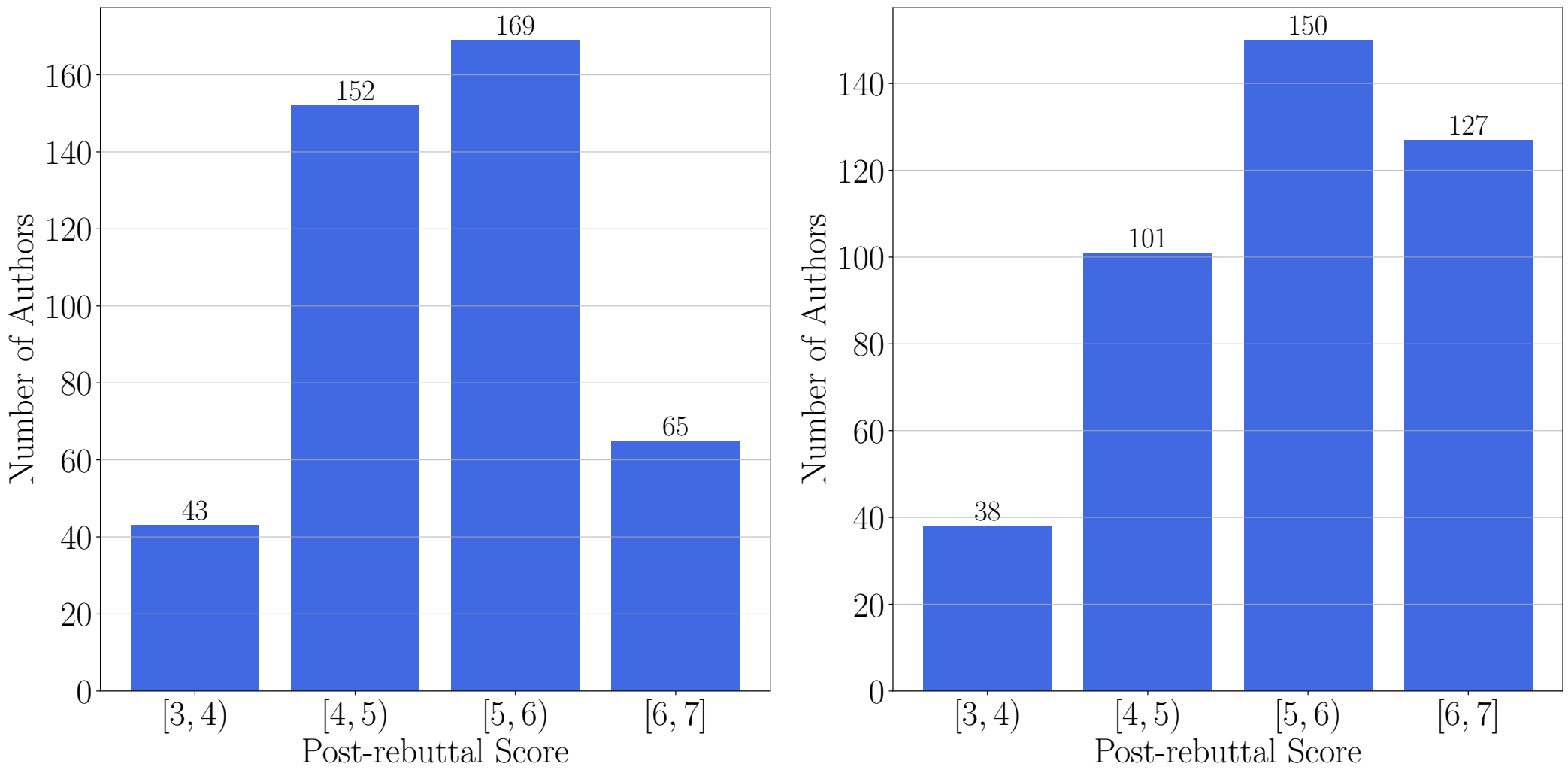

Supplementary Figure 4: Sample sizes by review-score interval. Bars and numerical labels show the number of responding authors with multiple ranked submissions in each interval; these are counts rather than derived summary statistics, so error bars and overlaid individual observations are not applicable. From left to right, the pre-rebuttal intervals contain $n = 43$, $n = 152$, $n = 169$ and $n = 65$ responding authors, and the post-rebuttal intervals contain $n = 38$, $n = 101$, $n = 150$ and $n = 127$ responding authors. No statistical test was applied.

base case, a no-reviewer-bias case, a no-score-noise case, a big-reviewer-bias case, and a big-score-noise case. Performance was measured by root mean squared error (RMSE), for which lower values indicate more accurate estimates of paper quality.

We matched the conference size and broad author/reviewer assignment structure to ICML 2023; the reviewer-bias and score-noise parameters described below retain the settings in the cited simulation design and were not estimated from the ICML 2023 data.

Let q_i denote the latent quality of paper i, b_r the additive bias of reviewer r, and σ_r the score-noise standard deviation of reviewer r. Before ranking-based adjustment, the raw score is generated as

$$\widetilde{S}_{ir} = q_i + b_r + \varepsilon_{ir}, \qquad \varepsilon_{ir} \mid \sigma_r \sim \mathcal{N}(0, \sigma_r^2),$$

where b_r and σ_r are shared across all papers assigned to reviewer r. Latent qualities are drawn from Gamma(shape $= 10$, scale $= 0.5$) and capped at 10. Each reviewer-side ranking is then sampled from a Plackett–Luce model with choice weights proportional to $\exp(q_i)$. The raw scores are isotonic-adjusted to agree with that ranking, clipped to $[1, 10]$, and rounded to integer scores.

The five scenarios use the following exact reviewer-level parameters; Gamma distributions are parameterized by shape and scale:

Scenario	Reviewer bias b_r	Noise standard deviation σ_r
Base	Uniform$[-2, 2]$	Gamma$(1, 1)$
No-Bias	0	Gamma$(1, 1)$
No-Var	Uniform$[-2, 2]$	0
Big-Bias	Uniform$[-3, 3]$	Gamma$(1, 1)$
Big-Var	Uniform$[-2, 2]$	Gamma$(1, 1.5)$

Relative to Base, Big-Bias changes only the bias distribution: its support is 50% wider, its standard deviation increases by 50%, and Var(b_r) increases from 4/3 to 3, a factor of 2.25. Big-Var changes only the distribution of the raw-score noise standard deviation: $\mathbb{E}(\sigma_r)$ increases

from 1 to 1.5, while the average conditional raw-score noise variance $\mathbb{E}(\sigma_r^2)$ increases from 2 to 4.5, also a factor of 2.25. Biases and noise standard deviations are expressed in review-score units, whereas their variances are expressed in squared review-score units; all refer to the pre-adjustment raw scores on the 1–10 scale. The label No-Var denotes $\sigma_r = 0$, which removes the additive Gaussian raw-score noise; the Plackett–Luce ranking remains stochastic.

Each paper was assigned to three or four reviewers. Latent qualities and assignment structures were fixed across scenarios, while reviewer parameters, raw-score noise, and Plackett–Luce rankings were redrawn in each of 10 repetitions. Thus, the standard deviations in Supplementary Table 5 describe across-repetition variation in RMSE, rather than standard errors or confidence intervals. This simulation is intended as a controlled mechanism and sensitivity check, not as an empirical estimate of the gains that would necessarily occur in ICML.

RMSE	Base	No-Bias	No-Var	Big-Bias	Big-Var
1. Average Scores (Benchmark)	0.850 (0.013)	0.589 (0.010)	0.693 (0.007)	1.077 (0.016)	0.948 (0.013)
2. Use Rankings from Reviewers	0.752 (0.014)	0.614 (0.006)	0.598 (0.005)	0.888 (0.016)	0.836 (0.012)
3. Use Rankings from Authors	0.787 (0.011)	0.555 (0.008)	0.650 (0.006)	0.984 (0.014)	0.872 (0.011)
4. Combine 2 and 3	0.709 (0.012)	0.585 (0.005)	0.571 (0.006)	0.829 (0.013)	0.782 (0.010)

Supplementary Table 5: Reviewer-ranking simulation. Columns identify the five synthetic bias and noise scenarios, and rows identify the four estimators of latent paper quality. Entries are mean root mean squared errors (RMSEs) across 10 repetitions, with standard deviations in parentheses; lower RMSE indicates more accurate recovery. The simulation follows the setup of Wang and Shi [2].

The results in Supplementary Table 5 show that reviewer-generated rankings can substantially reduce RMSE when reviewer biases are present, and that combining reviewer and author rankings often performs best. In the base case, RMSE decreases from 0.850 for average review scores to 0.752 when using reviewer rankings, 0.787 when using author rankings, and 0.709 when combining reviewer and author rankings. In the big-bias case, RMSE decreases from 1.077 for average review scores to 0.888 with reviewer rankings, 0.984 with author rankings, and 0.829 when combining both sources. The no-bias case is the main exception: when reviewer bias is absent, average scores already perform well and reviewer rankings do not improve RMSE. This result is complementary to our study: reviewer rankings are promising for score calibration and bias correction, whereas our analysis provides empirical evidence that author self-rankings contain externally validated information about later citation impact. Future review-system designs may benefit from combining these two comparative signals, while accounting for their different sources and limitations.

Supplementary Section 6. LLM-based pairwise rankings

To situate author self-rankings relative to recent pairwise-comparison approaches to peer review, we conducted a complementary analysis following the setup of Zhang et al. [3]. Because rejected-paper PDFs are not publicly available, this analysis was limited to accepted ICML 2023 submissions with downloadable PDFs. For each author with at least two accepted submissions and distinct author-provided ranks, we selected the author's highest-ranked and lowest-ranked accepted papers, yielding 263 author-level pairs. We extracted the title, abstract, figure/table captions, and main text using the ScienceBeam preprocessing pipeline, and asked GPT-4o mini with temperature 0 to choose the stronger paper using the pairwise-comparison prompt format of Zhang et al. [3]. We disabled web search and provided only the extracted paper content, preventing the model from accessing OpenReview scores, decisions, or other external information.

Supplementary Figure 5 compares the citation trajectories implied by author self-rankings and GPT pairwise rankings on the same set of paper pairs. Author self-rankings showed a clear citation signal: the author-highest-ranked accepted papers received 27.00 citations on average, compared with 13.82 for the author-lowest-ranked accepted papers. In contrast, GPT's preferred papers received 21.69 citations on average, compared with 19.61 for GPT's non-preferred papers, and this difference was not statistically significant. Thus, while LLM pairwise comparisons contain some directional signal in this constrained text-only setting, they recover less of the author-ranking signal than the authors' own comparative judgments.

This comparison should be interpreted as complementary to Zhang et al. [3], not as a full replication of their large-scale setting. Their study evaluates large-scale LLM pairwise comparisons across a broad submission pool, whereas our diagnostic comparison is restricted to within-author accepted-paper pairs for which PDFs were available. LLM-based rankings may perform better with the full submission pool, more capable models, and retrieval or web search enabled. The result nevertheless reinforces the main message of this paper: comparative judgments can be useful for peer review, but the source of the comparison matters; authors' self-rankings appear to capture information about the relative promise of their own work that is not fully recovered from paper text alone.

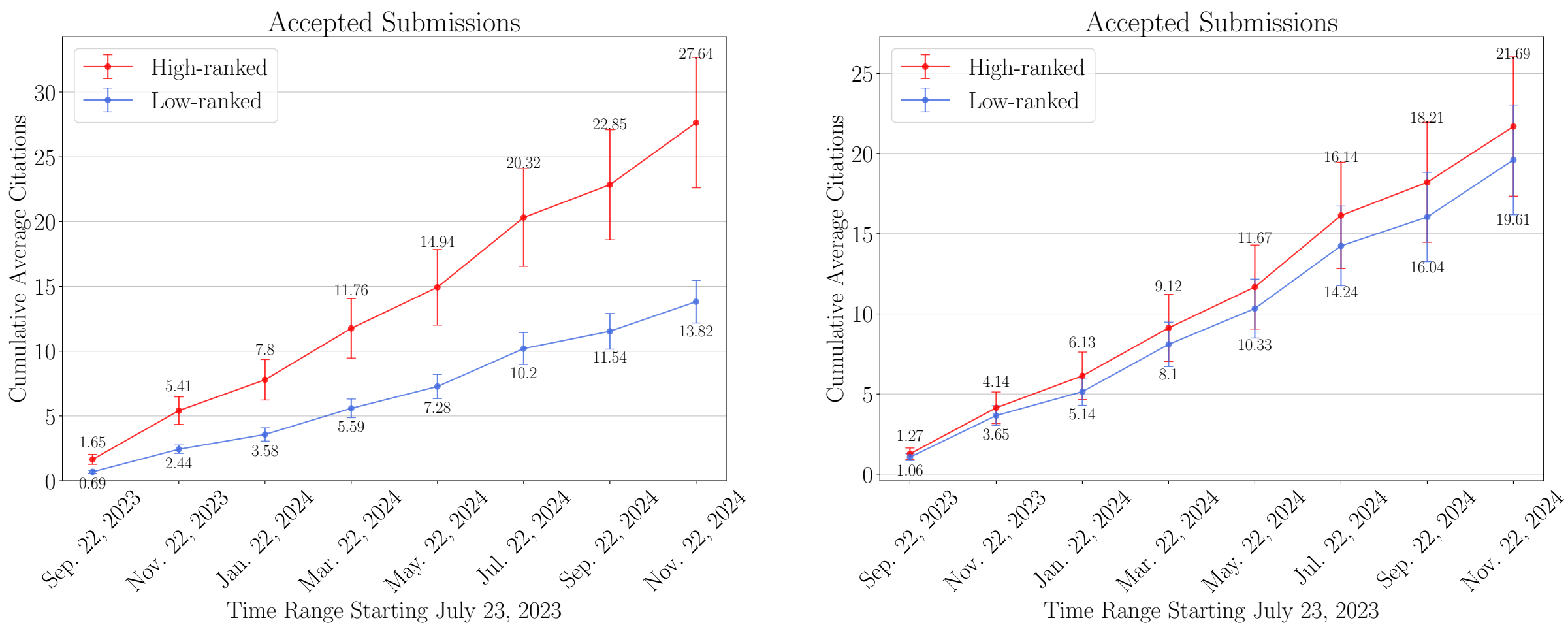

(a) Author-ranking comparison

(b) GPT-ranking comparison

Supplementary Figure 5: Cumulative citation trajectories from author and GPT pairwise rankings. The left panel compares author high- and low-ranked accepted submissions in the full accepted-author sample ($n = 280$ responding-author pairs per curve). The right panel compares submissions selected and not selected by GPT-4o mini among pairs with a valid model choice ($n = 263$ responding-author pairs per curve). Curves show mean cumulative Semantic Scholar citations at successive two-month endpoints; error bars show standard errors of the mean (SEM). Red and royal-blue curves identify the paired groups shown in each panel's legend. At the final endpoint, the left-panel comparison used a two-sided paired Wilcoxon signed-rank test ($P = 0.006$), and the right-panel comparison used a two-sided paired permutation test of the mean difference with 100,000 sign permutations ($P = 0.711$).

Supplementary Section 7. Additional confounder checks

Supplementary Figure 6 presents the arXiv posting dates for high- and low-ranked papers, along with replications of Figures 1 and 2 of the main text, excluding self-citations between July 23,

2023, and November 22, 2024. We observe that the distribution of arXiv posting times is similar between the high- and low-ranked groups, suggesting that the timing of arXiv release is reasonably balanced. Moreover, our main results continue to hold after excluding self-citations.

We also note that, in the upper right panel of Supplementary Figure 6, to quantify the posting time, we converted each arXiv posting datetime into the number of seconds since January 1, 1970 at 00:00:00 Coordinated Universal Time (UTC), commonly known as the UNIX timestamp. A Welch's t-test comparing the UNIX timestamp in two groups yields a p-value of 0.72, suggesting that arXiv posting time does not significantly differ between groups and is unlikely to confound our analysis. Moreover, a higher proportion of the low-ranked papers are posted either very early or very late compared to the high-ranked papers. This could be attributed to differences in submission quality. Low-ranked papers are more likely to have been rejected from prior venues and later recycled for submission to ICML 2023 (see Table 1 in [4]). Similarly, papers posted very late on arXiv often correspond to submissions that were rejected from ICML 2023 and later resubmitted to subsequent AI conferences. Finally, we make an observation related to the adversarial aspects of the double-blind review process. Notably, over 25% of submissions were posted on arXiv before the paper submission deadline, and over 50% were posted prior to the review release to authors. Such early postings may introduce bias into the review process and pose challenges to maintaining authors' anonymity.

Supplementary Section 8. Alternative impact metrics

To further examine how self-rankings align with paper quality and impact, we consider GitHub Stars as an alternative metric. We compare high- and low-ranked papers in terms of the GitHub Stars they received.

GitHub stars. Among the 2,530 submissions with valid rankings, 678 submissions were matched to a GitHub repository with valid GitHub Star counts. This set includes 232 authors with multiple ranked submissions and valid GitHub data. Using the same grouping approach as before, we find that high-ranked and accepted papers received an average of 755.15 GitHub Stars, more than twice that of low-ranked and accepted papers, which averaged 347.48 Stars. A similar trend is observed among rejected submissions. High-ranked and rejected papers received 89.77 GitHub Stars on average, while low-ranked and rejected papers received 33.27 Stars.

Google Scholar citations. We further validate the main findings on identifying well-cited papers and comparing self-rankings with review scores by repeating the analysis using citation counts from Google Scholar. Since Google Scholar does not support filtering by date range and does not exclude self-citations, we restrict the analysis to those presented in Extended Data Table 1, Extended Data Figure 2, and Figure 2 of the main text. The corresponding results using Google Scholar are reported in Supplementary Table 6, Supplementary Figure 7 and Supplementary Figure 8. We find that the patterns are highly similar to those observed using citation counts from Semantic Scholar. One notable difference is that the citation counts in Supplementary Table 6 are higher than those in Extended Data Table 1. This is expected, as the Google Scholar counts are aggregated from the paper's initial arXiv posting up to November 22, 2024, while the Semantic Scholar data in Supplementary Table 1 only includes citations from July 23, 2023 to November 22, 2024.

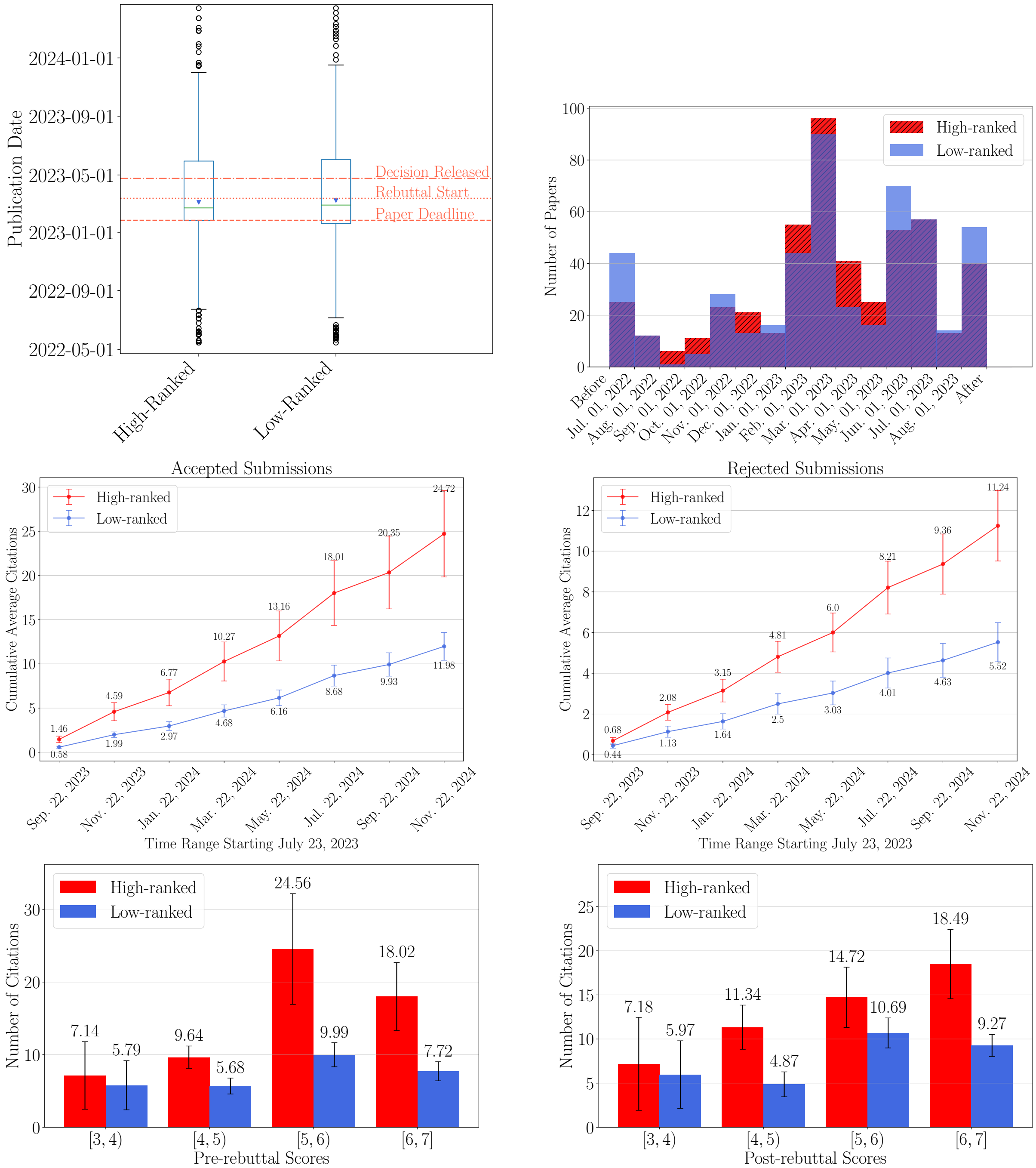

Supplementary Figure 6: Posting-time and non-self-citation analyses. **Upper**: arXiv posting-date box plot and histogram (high-ranked, $n = 501$; low-ranked, $n = 499$ author observations). Red lines mark submission, review and decision dates. Boxes show the median (centre) and 25th–75th percentiles (bounds); whiskers extend to 1.5 times the interquartile range and points lie beyond them. Two-sided Welch's t-test: $P = 0.72$. **Middle**: mean cumulative non-self-citations (accepted, $n = 280$ author pairs per curve; rejected or withdrawn, $n = 343$). **Lower**: mean non-self-citations in intervals [3,4), [4,5), [5,6) and [6,7]; per-rank-group $n = 43/152/169/65$ before and $n = 38/101/150/127$ after rebuttal. For the middle and lower rows, red denotes high-ranked and royal-blue low-ranked groups; error bars show standard errors of the mean (SEM).

		Citation Count for Accepted Papers			Citation Count for Rejected Papers		
		Rank	Post-Rebuttal Score	Pre-Rebuttal Score	Rank	Post-Rebuttal Score	Pre-Rebuttal Score
Mean	High	41.57	39.20	36.13	16.40	14.85	15.35
	Low	21.25	26.46	27.97	10.24	13.54	11.55
p-value		6.3×10^{-3}	0.10	0.29	1.0×10^{-2}	0.67	0.18
Max	High	1462.0	880.0	880.0	539.0	516.0	539.0
	Low	485.0	1462.0	1462.0	516.0	539.0	516.0
75th percentile	High	33.5	33.5	31.5	12.0	10.0	11.0
	Low	24.0	25.5	26.0	9.0	10.0	9.0

Supplementary Table 6: Google Scholar citation summary by ranking measure and final decision. Columns separate author ranking, post-rebuttal score and pre-rebuttal score comparisons within accepted and rejected submissions. Rows report group means, paired t-test P-values, maxima and 75th percentiles for citations accumulated from each paper's initial arXiv posting through November 22, 2024.

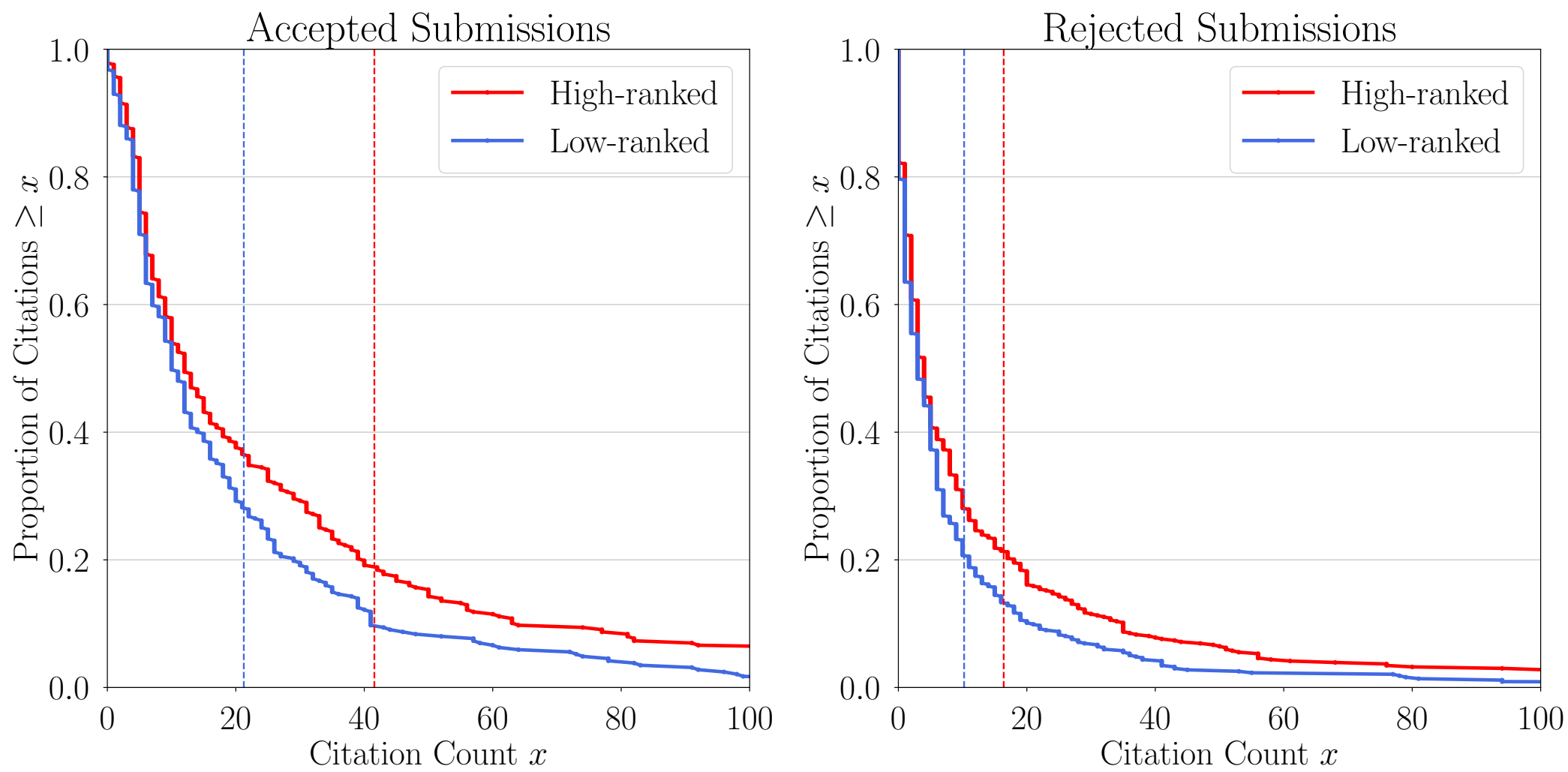

Supplementary Figure 7: Google Scholar citation distributions by author ranking and final decision. Curves show the empirical complementary cumulative distribution, with the horizontal axis giving citation threshold x and the vertical axis giving the proportion of submissions with more than x citations. The left panel contains $n = 280$ responding-author pairs of accepted submissions per rank group, and the right panel contains $n = 343$ responding-author pairs of rejected submissions per rank group. Red and royal-blue curves denote high- and low-ranked submissions, respectively; vertical dashed lines in the corresponding colours mark group means. Two-sided paired t-tests comparing high- and low-ranked citation counts yielded $P = 0.0063$ for accepted submissions and $P = 0.010$ for rejected submissions.

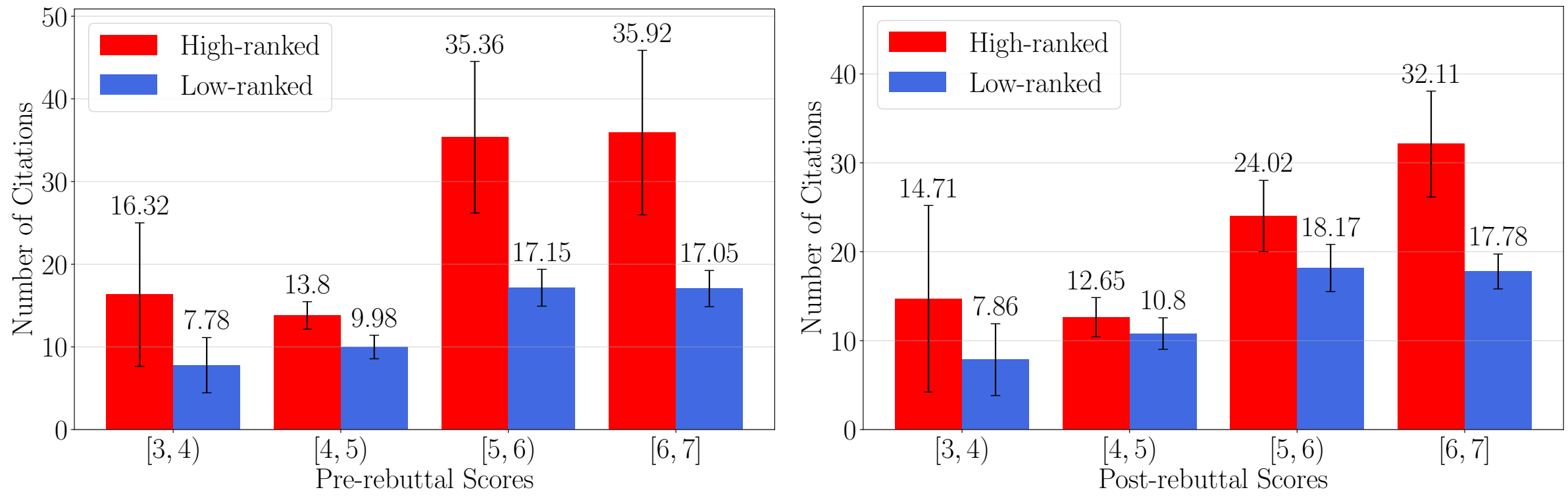

Supplementary Figure 8: Google Scholar citations by author ranking within review-score intervals. Red and royal-blue bars show mean citations accumulated from each paper's initial arXiv posting through November 22, 2024, for high- and low-ranked submissions, respectively; error bars show standard errors of the mean (SEM). From left to right, the pre-rebuttal intervals contain $n = 43$, $n = 152$, $n = 169$ and $n = 65$ responding-author pairs per rank group, and the post-rebuttal intervals contain $n = 38$, $n = 101$, $n = 150$ and $n = 127$ responding-author pairs per rank group. Individual author-level observations are not displayed because the underlying ranking data are private. No statistical comparison is displayed in this figure.

Supplementary Section 9. Distributional view of cumulative citations

Supplementary Figure 9 shows the distributions underlying the cumulative time-series panels of Figure 1 in the main text. This distributional view complements the means and standard errors shown in the main figure while retaining the same author-level paired comparisons.

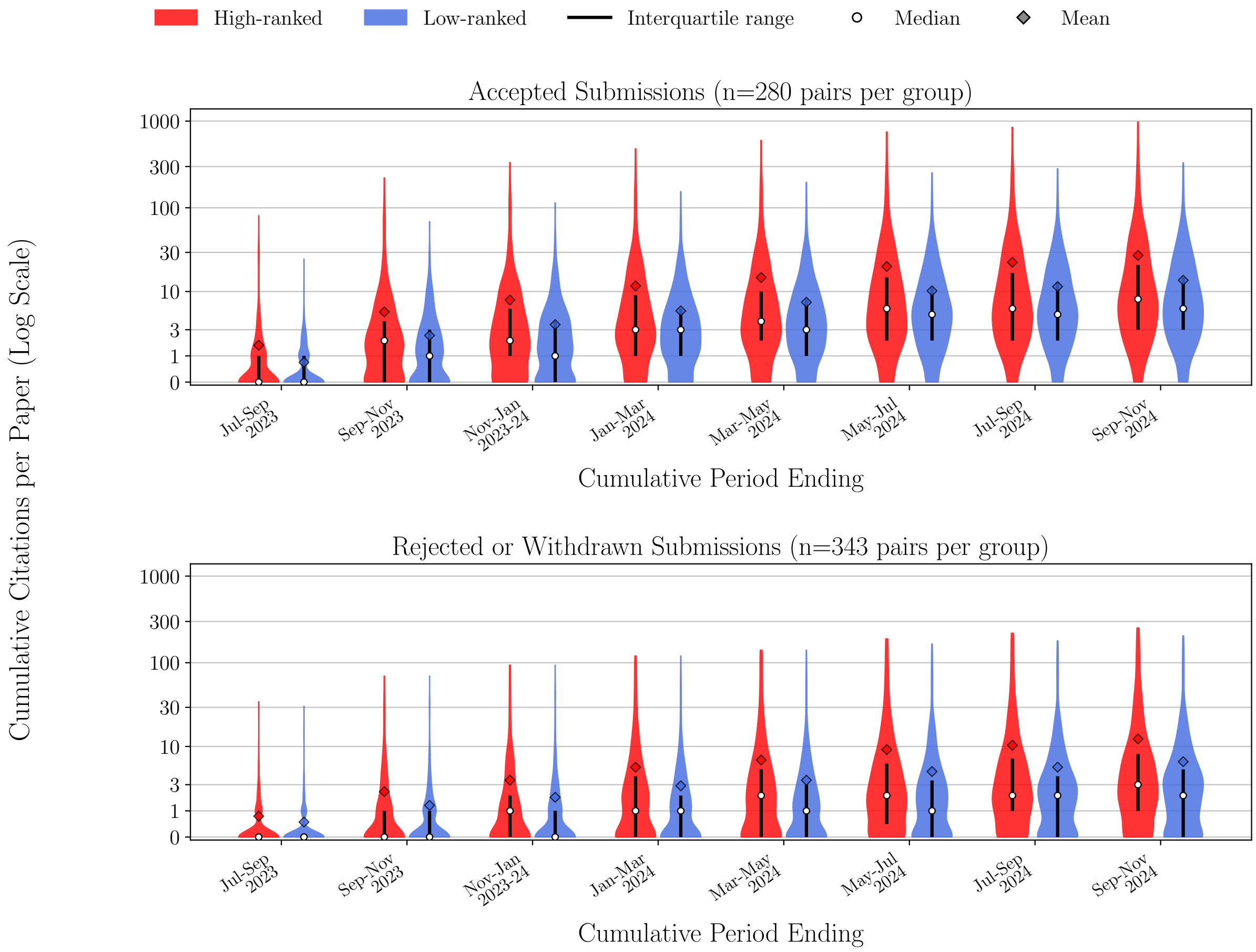

Supplementary Figure 9: Cumulative citation-count distributions underlying Figure 1 of the main text. **Upper**: accepted submissions. **Lower**: rejected or withdrawn submissions. Violin widths show Gaussian kernel density after transforming counts as $\log(1 + x)$, where x is the cumulative citation count; Scott's rule determines the bandwidth. Vertical-axis tick labels give cumulative citation counts on the original scale. Red violins show high-ranked submissions and royal-blue violins show low-ranked submissions. Black vertical lines span the 25th to 75th percentiles, white circles denote medians, and diamonds denote arithmetic means on the original cumulative citation-count scale. Each rank group contains $n = 280$ author-level observations in the accepted panel and $n = 343$ in the rejected or withdrawn panel. The unit of analysis is the responding author: within each final-decision group, each author contributes one highest-ranked and one lowest-ranked submission, forming a paired comparison in which the low-ranked submission is the within-author comparator. The stated n values count author-level pairs rather than unique papers; a submission can occur in more than one pair when multiple coauthors responded.

References

[1] Jake M Hofman, Duncan J Watts, Susan Athey, Filiz Garip, Thomas L Griffiths, Jon Kleinberg, Helen Margetts, Sendhil Mullainathan, Matthew J Salganik, Simine Vazire, Alessandro Vespignani, and Tal Yarkoni. Integrating explanation and prediction in computational social science. *Nature*, 595(7866):181–188, 2021. doi: 10.1038/s41586-021-03659-0.

[2] Weichen Wang and Chengchun Shi. From authors to reviewers: Leveraging rankings to improve peer review. *arXiv preprint arXiv:2510.21726*, 2025.

[3] Yaohui Zhang, Haijing Zhang, Wenlong Ji, Tianyu Hua, Nick Haber, Hancheng Cao, and Weixin Liang. From replication to redesign: Exploring pairwise comparisons for llm-based peer review. *Advances in Neural Information Processing Systems*, 38:11778–11805, 2026.

[4] Buxin Su, Jiayao Zhang, Natalie Collina, Yuling Yan, Didong Li, Kyunghyun Cho, Jianqing Fan, Aaron Roth, and Weijie Su. The ICML 2023 ranking experiment: Examining author self-assessment in ML/AI peer review. *Journal of the American Statistical Association*, pages 1–16, 2025. Just accepted.
