# Supplementary material for: Self-rankings as a predictor of scientific impact beyond peer review
Source: Nat Comput Sci. 2026 Aug 24;6(9):939–44. doi: 10.1038/s43588-026-01039-0 (PMC13593569; doi:10.1038/s43588-026-01039-0)
Supplement: Supplementary file 2 [file 43588_2026_1039_MOESM2_ESM.pdf]

nature portfolio

Corresponding author(s): Bingxin Zhao and Weijie Su

Last updated by author(s): Jul 22, 2026

# Reporting Summary

Nature Portfolio wishes to improve the reproducibility of the work that we publish. This form provides structure for consistency and transparency in reporting. For further information on Nature Portfolio policies, see our Editorial Policies and the Editorial Policy Checklist.

## Statistics

For all statistical analyses, confirm that the following items are present in the figure legend, table legend, main text, or Methods section.

| n/a | Confirmed | |
|---|---|---|
| ☐ | ☒ | The exact sample size (*n*) for each experimental group/condition, given as a discrete number and unit of measurement |
| ☐ | ☒ | A statement on whether measurements were taken from distinct samples or whether the same sample was measured repeatedly |
| ☐ | ☒ | The statistical test(s) used AND whether they are one- or two-sided<br>*Only common tests should be described solely by name; describe more complex techniques in the Methods section.* |
| ☐ | ☒ | A description of all covariates tested |
| ☐ | ☒ | A description of any assumptions or corrections, such as tests of normality and adjustment for multiple comparisons |
| ☐ | ☒ | A full description of the statistical parameters including central tendency (e.g. means) or other basic estimates (e.g. regression coefficient) AND variation (e.g. standard deviation) or associated estimates of uncertainty (e.g. confidence intervals) |
| ☐ | ☒ | For null hypothesis testing, the test statistic (e.g. *F*, *t*, *r*) with confidence intervals, effect sizes, degrees of freedom and *P* value noted<br>*Give P values as exact values whenever suitable.* |
| ☒ | ☐ | For Bayesian analysis, information on the choice of priors and Markov chain Monte Carlo settings |
| ☐ | ☒ | For hierarchical and complex designs, identification of the appropriate level for tests and full reporting of outcomes |
| ☒ | ☐ | Estimates of effect sizes (e.g. Cohen's *d*, Pearson's *r*), indicating how they were calculated |

*Our web collection on statistics for biologists contains articles on many of the points above.*

## Software and code

Policy information about availability of computer code

| | |
|---|---|
| Data collection | Data were collected in two phases. In Phase 1, submission metadata, reviewer scores, and final decisions for ICML 2023 were obtained from OpenReview, which hosted the conference review process. Author self-rankings were collected using OpenRank.cc, a custom platform developed for this study and deployed as a survey to authors after the submission deadline. In Phase 2, citation data were retrieved from Semantic Scholar via its API, and records were matched to ICML 2023 submissions using custom title- and author-matching procedures based on Levenshtein distance. For supplementary robustness analyses, Google Scholar citation counts and GitHub star counts were also collected where available. The custom data-collection code is publicly available at https://github.com/BuxinSu/ICML_2023_Citation and archived as version v1.0.0 at https://doi.org/10.5281/zenodo.21501732. |
| Data analysis | Data cleaning, record linkage, statistical analysis, and figure/table generation were performed using custom code developed by the authors. This code was used to validate title and author matches, construct the analytic dataset, compute citation-based summary statistics, perform paired t-tests and Welch's t-tests, stratify analyses by acceptance status and review-score groups, and run sensitivity analyses excluding self-citations. No additional commercial analysis software is specified in the manuscript. The custom data-processing, statistical-analysis and figure-generation code is publicly available at https://github.com/BuxinSu/ICML_2023_Citation and archived as version v1.0.0 at https://doi.org/10.5281/zenodo.21501732. |

For manuscripts utilizing custom algorithms or software that are central to the research but not yet described in published literature, software must be made available to editors and reviewers. We strongly encourage code deposition in a community repository (e.g. GitHub). See the Nature Portfolio guidelines for submitting code & software for further information.

## Data

Policy information about availability of data

All manuscripts must include a data availability statement. This statement should provide the following information, where applicable:

- Accession codes, unique identifiers, or web links for publicly available datasets
- A description of any restrictions on data availability
- For clinical datasets or third party data, please ensure that the statement adheres to our policy

The study used: (1) ICML 2023 submission, author-ranking, reviewer-score and decision records collected through OpenReview and OpenRank.cc; (2) Semantic Scholar citation records covering 23 July 2023 to 22 November 2024; (3) Google Scholar citation counts collected through SerpAPI; and (4) GitHub star counts for matched repositories. The study generated a linked author–submission–review–citation dataset and privacy-preserving aggregate Source Data.

Individual-level data, including author self-rankings and linked submission and citation records, cannot be made publicly available or shared upon request because of the ICML 2023 Board policy and the privacy terms under which the data were collected (see https://openrank.cc/legal/privacy/). No controlled-access mechanism is available for these data. Privacy-preserving aggregate summary statistics underlying the figures are provided as Source Data with this paper. These files contain no individual-level observations, author or submission identifiers, or author–submission pairings. No additional individual-level source data are available.

## Research involving human participants, their data, or biological material

Policy information about studies with human participants or human data. See also policy information about sex, gender (identity/presentation), and sexual orientation and race, ethnicity and racism.

| | |
|---|---|
| Reporting on sex and gender | No sex- or gender-related data were collected or analyzed in this study, and no sex- or gender-based analyses were performed. The study focused on authors of ICML 2023 submissions and their self-rankings, review scores, acceptance decisions, and citation outcomes. |
| Reporting on race, ethnicity, or other socially relevant groupings | No data on race, ethnicity, or other socially relevant groupings were collected or analyzed in this study, and no analyses were stratified by such variables. The variables analyzed were authors' submission rankings, reviewer scores, conference decisions, and citation-based outcomes. |
| Population characteristics | The human participants were researchers/authors who submitted papers to ICML 2023. Phase One involved the ICML 2023 author pool of 18,535 authors across 6,538 submissions. An official survey sent through OpenReview was completed by 5,634 authors, and 1,342 authors with multiple submissions provided valid rankings. The final citation-analysis dataset included 797 authors with multiple ranked submissions and valid citation data, corresponding to 1,527 unique submissions. No demographic or clinical population characteristics such as sex, gender, race, ethnicity, age, diagnosis, or treatment status were collected. |
| Recruitment | All 18,535 ICML 2023 authors were invited to participate through an official survey distributed via OpenReview. Participation was voluntary. Self-selection bias may therefore be present because respondents may differ from nonrespondents in their engagement with the review process, confidence in their submissions or willingness to disclose rankings. The primary analysis also excluded authors with only one submission and records without valid rankings or citation matches. These restrictions limit representativeness and generalizability and may affect the estimated effect sizes. The findings are therefore interpreted as associations among participating authors with multiple submissions, rather than as estimates applicable to all ICML authors or other research communities. |
| Ethics oversight | The experiment was designed and implemented at ICML 2023 with the approval of the University of Pennsylvania IRB Team and conference organizers. |

Note that full information on the approval of the study protocol must also be provided in the manuscript.

# Field-specific reporting

Please select the one below that is the best fit for your research. If you are not sure, read the appropriate sections before making your selection.

☐ Life sciences ☒ Behavioural & social sciences ☐ Ecological, evolutionary & environmental sciences

For a reference copy of the document with all sections, see nature.com/documents/nr-reporting-summary-flat.pdf

# Behavioural & social sciences study design

All studies must disclose on these points even when the disclosure is negative.

| | |
|---|---|
| Study description | This was a quantitative, observational study combining an online survey-based experiment with administrative peer-review records and bibliometric follow-up. In Phase One, ICML 2023 authors with multiple submissions were asked to self-rank their papers by perceived quality/significance. In Phase Two, these ranked submissions were linked to subsequent citation outcomes, and analyses compared higher- versus lower-ranked papers within authors and against review scores and final decisions. |

| | |
|---|---|
| Research sample | The study combined ICML 2023 submission metadata, review scores and final decisions from OpenReview; author self-rankings collected through OpenRank.cc; and citation records from Semantic Scholar. Google Scholar citations and GitHub star counts were used in supplementary analyses. The sampling frame comprised 18,535 authors associated with 6,538 ICML 2023 submissions. The survey was completed by 5,634 authors (30.4%), of whom 1,342 authors with multiple submissions provided valid rankings covering 2,592 submissions. The primary citation analysis included 797 authors and 1,527 unique submissions with valid rankings and citation matches. No age, sex, gender, race, ethnicity or other demographic information was collected. The sample is not representative of all ICML 2023 authors because participation was voluntary and the analysis required multiple submissions, valid rankings and successful citation matching. This sample was selected because within-author comparisons require at least two ranked submissions and observable citation outcomes. |
| Sampling strategy | This was not a random sample. Rather, the study used a census-style invitation to all ICML 2023 authors through OpenReview, with the core analytic sample consisting of authors who had multiple submissions and chose to complete the survey. Sample sizes were determined by the size of the ICML 2023 submission pool, voluntary participation, the availability of valid rankings, and successful downstream citation matching. |
| Data collection | Self-rankings were collected through an online, self-administered survey implemented on OpenRank.cc and distributed through OpenReview immediately after the ICML 2023 submission deadline. Authors ranked their submissions by dragging them into order and completed additional questionnaire items. Review scores and final decisions were obtained from OpenReview. Citation records were collected through the Semantic Scholar API and matched to submissions using title and author information and Levenshtein distance; Google Scholar citations and GitHub star counts were collected for supplementary analyses. No researcher or other person was present during survey completion. Blinding to experimental condition was not applicable because the study was observational and involved no intervention or group allocation. Researchers were not blinded to the study hypothesis; however, author rankings were collected before review outcomes and subsequent citation outcomes were observed. |
| Timing | The survey was sent on January 26, 2023, immediately after the submission deadline, and closed on February 10, 2023. Citation follow-up then covered papers citing the ranked submissions between July 23, 2023 and November 22, 2024. Thus, data collection occurred in two phases: survey collection in late January to early February 2023, followed by bibliometric outcome collection over the subsequent citation window. |
| Data exclusions | Data were excluded from the primary citation analyses when submissions could not be validly matched to Semantic Scholar records under the manuscript's title/author matching procedure. Starting from 1,342 authors with multiple ranked submissions and 2,592 ranked submissions, the final primary citation dataset included 797 authors and 1,527 unique submissions. |
| Non-participation | Of the 18,535 ICML 2023 authors invited, 5,634 completed the survey and 12,901 did not, corresponding to a 30.4% response rate. At the submission level, 2,592 submissions were ranked by at least one author, meaning 3,946 submissions were not represented in the ranking data. |
| Randomization | Participants were not allocated to experimental groups, and no randomization was used. |

# Reporting for specific materials, systems and methods

We require information from authors about some types of materials, experimental systems and methods used in many studies. Here, indicate whether each material, system or method listed is relevant to your study. If you are not sure if a list item applies to your research, read the appropriate section before selecting a response.

## Materials & experimental systems

| n/a | Involved in the study |
|---|---|
| ☒ | ☐ Antibodies |
| ☒ | ☐ Eukaryotic cell lines |
| ☒ | ☐ Palaeontology and archaeology |
| ☒ | ☐ Animals and other organisms |
| ☒ | ☐ Clinical data |
| ☒ | ☐ Dual use research of concern |
| ☒ | ☐ Plants |

## Methods

| n/a | Involved in the study |
|---|---|
| ☒ | ☐ ChIP-seq |
| ☒ | ☐ Flow cytometry |
| ☒ | ☐ MRI-based neuroimaging |

## Plants

| | |
|---|---|
| Seed stocks | NA |
| Novel plant genotypes | NA |
| Authentication | NA |
