# Supplementary material for: Self-rankings as a predictor of scientific impact beyond peer review
Source: Nat Comput Sci. 2026 Aug 24;6(9):939–44. doi: 10.1038/s43588-026-01039-0 (PMC13593569; doi:10.1038/s43588-026-01039-0)
Supplement: Supplementary file 3 [file 43588_2026_1039_MOESM3_ESM.pdf]

nature portfolio

Peer Review File

Self-Rankings as a Predictor of Scientific Impact Beyond Peer Review

Corresponding Author: Dr Weijie Su

Version 0:

Reviewer comments:

Reviewer #1

(Remarks to the Author)
This manuscript addresses an important and timely question for scientific evaluation: whether authors' comparative assessments of their own submissions contain useful signal beyond conventional peer review. The study is ambitious in scale and, in my view, convincingly executed. In particular, the participation is notable for a field of this size: the authors obtained rankings from 1,342 researchers covering 2,592 submissions, corresponding to 39.6% of all ICML 2023 submissions. The paper is also strengthened by the effort to follow outcomes longitudinally and by the clear empirical finding that author self-rankings are strongly associated with later citation impact.

The results are both interesting and potentially important for future review systems. Papers ranked highest by their authors received about twice as many citations as the lowest-ranked papers, and self-rankings predicted future citations better than pre- or post-rebuttal review scores. These findings suggest that author self-rankings may offer a useful, low-cost complementary signal of downstream impact.

I am overall positive about the manuscript. My main suggestion is not a concern about validity, but rather an opportunity to better situate the contribution within the emerging literature on comparative review mechanisms. It would also be valuable to position this work relative to recent efforts on pairwise-comparison-based review, especially Zhang et al., From Replication to Redesign: Exploring Pairwise Comparisons for LLM-Based Peer Review (NeurIPS 2025), which studies large-scale LLM-based pairwise ranking as an alternative to conventional paper scoring. That study similarly replaces absolute scoring with pairwise comparisons and evaluates selection quality using later citations, but uses large-scale LLM comparisons rather than author self-rankings. It reports near-human selection performance at scale, while also finding reduced topic novelty and greater institutional concentration among selected papers.

Against that backdrop, the present manuscript offers an especially valuable result: human self-rankings appear to provide a strong signal of future impact, and in this study they outperform the official review scores. I would therefore encourage the authors to discuss more explicitly what this implies for the broader debate on AI-assisted peer review. At a minimum, the paper could articulate more clearly that comparative judgments seem promising, but that the source of those judgments may matter substantially, that human self-rankings may carry a different, and perhaps stronger, signal than LLM-based rankings. A particularly informative extension would be a direct comparison between author self-rankings and LLM-based pairwise rankings on the same submission pool, for example using the framework of Zhang et al. Concretely, the authors could run 10^5-10^6 LLM pairwise comparisons, measure agreement with the authors' self-rankings, and compare the citation outcomes and qualitative characteristics of the papers prioritized by each method. Such an analysis could help clarify whether LLM rankings mainly favor more conventional, well-executed work, whereas author rankings better surface less conventional or potentially field-shaping contributions.

Overall, this is a strong, carefully conducted, and practically relevant study. The main claims are well supported, and the work has clear implications for the future design of peer review in fast-growing fields. I am supportive of publication.

(Remarks on code availability)
I reviewed the repository materials but did not run the code myself. The analysis appears to be relatively simple and largely statistical, so the main results should be reproducible in principle provided the underlying data and dependencies are available.

Reviewer #2

(Remarks to the Author)
This paper examines whether authors' self-rankings of their own multiple submissions can help identify high-impact AI papers. Using an ICML 2023 field setting, the authors asked researchers with multiple submissions to rank their papers shortly after submission and before final review outcomes. They then linked these rankings to later citation data and found that authors' top-ranked papers received substantially more citations than their lowest-ranked papers. The paper's main contribution is to show that author self-rankings contain useful predictive information about future citation impact and may provide a low-cost complement to traditional peer review.

#Strengths

The paper has several important strengths. It addresses a timely and practically significant problem: peer review is under strain from rapid submission growth, reviewer overload, and noisy evaluation. Its empirical setting is strong because the authors collected self-rankings from actual ICML 2023 authors immediately after submission and before review outcomes, making the study closely connected to real conference practice. The within-author design is also compelling, as comparing an author's own top-ranked and bottom-ranked submissions helps control for author-level differences such as reputation, research area, productivity, and expertise. Substantively, the finding that author self-rankings predict future citation impact, and appear to do so better than pre- or post-rebuttal review scores, is both (somewhat) surprising and practically useful. The paper is further strengthened by robustness checks that stratify by acceptance decision, compare papers with similar review scores, and address potential confounds such as preprint timing and self-citations.

#Weakness/ Questions

A central limitation is that the paper's main evidence appears to come from an extreme top-vs-bottom comparison rather than from the full ranking. This raises several questions about interpretation and practical use. The current analysis shows that authors' top-ranked papers tend to receive more citations than their lowest-ranked papers, but this is a weaker and narrower claim than showing that authors' complete ordinal rankings are informative. In other words, the paper demonstrates that authors may be able to identify their perceived best and worst submissions, but it does not yet fully establish that they can meaningfully distinguish among all submissions in between.

First, papers ranked in the middle are largely excluded from the headline analysis, so it remains unclear whether ranks such as #2, #3, or #4 contain useful predictive information. This matters for implementation: if a conference were to incorporate author rankings into peer review, it would need guidance on how to interpret the entire ranking, not only the first and last positions. The authors should therefore test whether citation impact declines monotonically across all ranking positions and whether adjacent ranks meaningfully differ from one another.

Second, the meaning of "low-ranked" differs across authors. For an author with two submissions, the low-ranked paper is simply second out of two; for an author with eleven submissions, it is eleventh out of eleven. These are quite different comparisons, but the high-vs-low design may treat them similarly. The authors should report heterogeneity by the number of submissions ranked, use normalized ranks, and examine whether the results are driven by authors with larger submission portfolios.

Third, multi-author papers complicate the classification because different coauthors may rank the same paper differently. If one coauthor ranks a paper first while another ranks it last, then "high-ranked" is not a stable paper-level property. Robustness checks using average rank across coauthors, only papers with consistent coauthor rankings, or rankings from primary/corresponding authors would help clarify whether the signal is robust.

Finally, the paper should clarify the exact survey prompt and explain whether authors were ranking perceived quality, expected acceptance, significance, personal contribution, or future impact. These criteria are related but not interchangeable, and the mechanism behind the result depends on what authors believed they were being asked to assess.

These issues do not undermine the core insight that author rankings contain useful information. However, how exactly self-rankings should be incorporated into peer review process needs more thoughts.

(Remarks on code availability)

Reviewer #3

(Remarks to the Author)
Very nice experiments! I would certainly welcome more experiments of this kind on the peer review process. That said, I have several major concerns about the current manuscript.

First, I wonder if the authors have invested sufficient effort in this study to merit a new publication, considering the earlier publications establishing the theoretical foundation of the experiment [1,2] and studies using data obtained from the same experiment [3,4]. I think later publications should be held to a higher standard, not lower, in demonstrating added value to science.

Admittedly, I can see that the focus of this paper is different from the earlier works, which led me to my second concern: I do not think Nature Computational Science is the right venue for this publication. Although the paper is certainly of interest to the machine learning community and the computer science community at large, the paper primarily relies on relatively basic bibliometric analyses of citation counts. For this reason, it seems that venues such as Scientometrics, Journal of Informetrics, or Research Policy are all better fits, compared to Nature Computational Science. The fact that the subject of study is a machine learning conference does not, by itself, make this venue the most appropriate outlet. Meanwhile, the type of analysis conducted here points to other venues as a better match.

Third, the motivation of this study is not clear to me. The authors state that “our study reveals that a powerful and overlooked source of insight is the authors themselves” but it is not clear to me who would benefit from this insight or how it should be used? The Discussion suggests that the method could help conferences evaluate and select manuscripts, yet the empirical analysis is not framed around that use case. For example, a conference would probably care about whether this self-reported ranking can be gamed by the authors (which you studied in your earlier work), they would also care about how accurate this signal is in terms of prediction and selection, but you do not describe how conferences are supposed to use this ranking information and neither do you report metrics such as precision and recall of any selection criteria (that a conference may use) relying on this metric. More fundamentally, even if you do come up with a use case employing this metrics, it is not clear to me that the primary concern of the conferences is to maximize citation count. Having high citation counts is arguably good for a conference, but to select papers based on its projected citation counts is very difficult to justify. For you to argue that the insights authors have about their own papers can be valuable to the conferences, you need alternative ways to evaluate the ranking information, in a way that more directly speaks to the quality of the underlying paper, than simply citation counts (or Github Stars).

Some comments about statistical tests
There lacks a justification of using t-tests throughout the paper (Are you using Welch’s t-test throughout the paper? This was not clear). Citation counts tend to be long-tailed. Depending on how severely long-tailed it is, t-test may not be the best choice.

Additionally, I think the authors should include an array of regression analyses with an increasing number of controls, and report the results in a big table (or plot of regression coefficients) just so that the readers can see how much the effect size (Cohen’s d or equivalent) changes when controlling for control variables. Also do report the R^2 and Akaike Information Criterion (AIC) or Bayesian Information Criterion (BIC) just so that we can see how much addition explanatory power the self-reported ranking carries, in addition to baseline models. Because you are dealing with non-negative count data, use Negative binomial regression and Poisson regression instead of OLS.

In addition to the above procedures that focuses on explaination, I also think it is useful to do the prediction analysis in the proper way, especially since authors use language such as "High rankings identify well-cited papers" and “Ranking is more predictive of citation counts” (for a discussion on explanation vs prediction, see https://doi.org/10.1038/s41586-021-03659-0) I would therefore like to see a proper predictive analysis using separate training and holdout sets, with evaluation metrics such as precision and recall.

One additional analysis may also be worth considering: Because each reviewer evaluates multiple papers, reviewers may implicitly provide ranking information across the papers they score. It would be interesting to examine whether that reviewer-generated ranking information can be incorporated into the analysis, just like the author-generated ranking information.

Finally, please include the IRB ethics approval information related to the experiment in the manuscript.

[1] https://pubsonline.informs.org/doi/10.1287/opre.2022.0622
[2] https://openreview.net/forum?id=xmx5rE9QP7R
[3] https://academic.oup.com/jrsssb/article-abstract/87/5/1422/8151400
[4] https://www.tandfonline.com/doi/abs/10.1080/01621459.2025.2510006

(Remarks on code availability)

Reviewer #4

(Remarks to the Author)
Very nice experiments! I would certainly welcome more experiments of this kind on the peer review process. That said, I have several major concerns about the current manuscript.

First, I wonder if the authors have invested sufficient effort in this study to merit a new publication, considering the earlier publications establishing the theoretical foundation of the experiment [1,2] and studies using data obtained from the same experiment [3,4]. I think later publications should be held to a higher standard, not lower, in demonstrating added value to science.

Admittedly, I can see that the focus of this paper is different from the earlier works, which led me to my second concern: I do not think Nature Computational Science is the right venue for this publication. Although the paper is certainly of interest to the machine learning community and the computer science community at large, the paper primarily relies on relatively basic bibliometric analyses of citation counts. For this reason, it seems that venues such as Scientometrics, Journal of Informetrics,

or Research Policy are all better fits, compared to Nature Computational Science. The fact that the subject of study is a machine learning conference does not, by itself, make this venue the most appropriate outlet. Meanwhile, the type of analysis conducted here points to other venues as a better match.

Third, the motivation of this study is not clear to me. The authors state that “our study reveals that a powerful and overlooked source of insight is the authors themselves” but it is not clear to me who would benefit from this insight or how it should be used? The Discussion suggests that the method could help conferences evaluate and select manuscripts, yet the empirical analysis is not framed around that use case. For example, a conference would probably care about whether this self-reported ranking can be gamed by the authors (which you studied in your earlier work), they would also care about how accurate this signal is in terms of prediction and selection, but you do not describe how conferences are supposed to use this ranking information and neither do you report metrics such as precision and recall of any selection criteria (that a conference may use) relying on this metric. More fundamentally, even if you do come up with a use case employing these metrics, it is not clear to me that the primary concern of the conferences is to maximize citation count. Having high citation counts is arguably good for a conference, but to select papers based on its expected citation counts is very difficult to justify. For you to argue that the insights authors have about their own papers can be valuable to the conferences, you need alternative ways to evaluate the ranking information, in a way that more directly speaks to the quality of the underlying paper, than simply relying on citation counts (or Github Stars).

Some comments about statistical tests
There lacks a justification of using t-tests throughout the paper (Are you using Welch’s t-test throughout the paper? This was not clear). Citation counts tend to be long-tailed. Depending on how severely long-tailed it is, t-test may not be the best choice.

Additionally, I think the authors should include an array of regression analyses with an increasing number of controls, and report the results in a big table (or plot of regression coefficients) just so that the readers can see how much the effect size (Cohen’s d or equivalent) changes when controlling for control variables. Also do report the R^2 and Akaike Information Criterion (AIC) or Bayesian Information Criterion (BIC) just so that we can see how much additional explanatory power the self-reported ranking carries, in addition to baseline models. Because you are dealing with non-negative count data, use Negative binomial regression and Poisson regression instead of OLS.

In addition to the above procedures that focuses on explanation, I also think it is useful to do the prediction analysis in the proper way, especially since authors use language such as "High rankings identify well-cited papers" and “Ranking is more predictive of citation counts” (for a discussion on explanation vs prediction, see https://doi.org/10.1038/s41586-021-03659-0) I would therefore like to see a proper predictive analysis using separate training and holdout sets, with evaluation metrics such as precision and recall.

One additional analysis may also be worth considering: Because each reviewer evaluates multiple papers, reviewers may implicitly provide ranking information across the papers they score. It would be interesting to examine whether that reviewer-generated ranking information can be incorporated into the analysis, just like the author-generated ranking information.

Finally, please include the IRB ethics approval information related to the experiment in the manuscript.

[1] https://pubsonline.informs.org/doi/10.1287/opre.2022.0622
[2] https://openreview.net/forum?id=xmx5rE9QP7R
[3] https://academic.oup.com/jrsssb/article-abstract/87/5/1422/8151400
[4] https://www.tandfonline.com/doi/abs/10.1080/01621459.2025.2510006

(Remarks on code availability)
Code is well-organized and well-documented. But since the authors did not provide the dataset being analyzed (understandably due to privacy concerns), it is not possible to execute the code and verify correctness. I suggest the authors release aggregated data to allow minimal replication of the results.

Version 1:

Reviewer comments:

Reviewer #1

(Remarks to the Author)
I have reviewed the revised manuscript and the authors’ response, and I find that they have satisfactorily addressed the points I raised in the previous round of review. In particular, the revision better situates the work within the broader literature on comparative review mechanisms and clarifies the role of author self-rankings as a complementary signal, so I remain supportive of publication.

(Remarks on code availability)

Reviewer #2

(Remarks to the Author)
Thanks for the resubmission. I appreciate the authors’ careful revisions and additional analyses. They have addressed all of my concerns and have clearly strengthened the manuscript.I recommend acceptance.

(Remarks on code availability)

Reviewer #3

(Remarks to the Author)
Thank you to the authors for the extensive revision. My previous concerns have been addressed. I have three additional questions regarding the revised manuscript:

1. The authors mention that they will add the paragraph beginning “Our work provides compelling evidence that authors’ self-rankings are a low-cost …” to the manuscript, but I am unable to locate it in the revised paper. The paragraph beginning “The purpose of the self-ranking experiment …” also appears to be missing. The latter may correspond to the highlighted paragraph on page 11, although the wording is different. However, I could not find anything similar to the former paragraph.

2. In Table S.4, the reported precision and recall appear to be low. How do the authors interpret this result?

3. For Table S.5, could the authors provide more technical details on the exact values of “big”-var and “big”-bias?

(Remarks on code availability)

Reviewer #4

(Remarks to the Author)
I co-reviewed this manuscript with one of the reviewers who provided the listed reports.

(Remarks on code availability)
I co-reviewed this manuscript with one of the reviewers who provided the listed reports.

Open Access This Peer Review File is licensed under a Creative Commons Attribution 4.0 International License, which permits use, sharing, adaptation, distribution and reproduction in any medium or format, as long as you give appropriate credit to the original author(s) and the source, provide a link to the Creative Commons license, and indicate if changes were made.
In cases where reviewers are anonymous, credit should be given to 'Anonymous Referee' and the source.
The images or other third party material in this Peer Review File are included in the article’s Creative Commons license, unless indicated otherwise in a credit line to the material. If material is not included in the article’s Creative Commons license and your intended use is not permitted by statutory regulation or exceeds the permitted use, you will need to obtain

permission directly from the copyright holder.
To view a copy of this license, visit https://creativecommons.org/licenses/by/4.0/
